# Per-Patient Polyp Detection Rate of Colon Capsule Endoscopy: A Systematic Review and Meta-Analysis with Exploratory Stratification by Referral Pathway

**DOI:** 10.3390/diagnostics16213493

**Published:** 2026-10-25

**Authors:** Ian Io Lei, Grazia Bernui Vigo, Ramesh P. Arasaradnam, Anastasios Koulaouzidis

**Affiliations:** 1 Institute of Precision Diagnostics & Translational Medicine, University Hospitals Coventry and Warwickshire NHS Trust, Coventry CV2 2DX, UK; 2 Department of Gastroenterology, Oxford University Hospitals NHS Foundation Trust, Oxford OX3 9DU, UK; 3 Department of Digestive Diseases, University Hospitals of Leicester NHS Trust, Leicester LE5 4PW, UK; 4 Leicester Cancer Centre, University of Leicester, Leicester LE1 7RH, UK; 5 Department of Clinical Research, University of Southern Denmark, 5230 Odense, Denmark; 6 Department of Surgery, Odense University Hospital, 5000 Odense, Denmark; 7 Department of Gastroenterology, Pomeranian Medical University, 70-204 Szczecin, Poland

**Keywords:** colon capsule endoscopy, polyp detection rate, diagnostic yield, referral pathway, systematic review, meta-analysis

## Abstract

**Background/Objectives**: Colon capsule endoscopy (CCE) is a minimally invasive alternative to optical colonoscopy, but per-patient polyp detection rate (PDR), the proportion of patients with at least one visually reported polyp, varies across referral populations. We estimated any-polyp and size-stratified CCE yield and explored between-study variation. **Methods**: MEDLINE, Embase, PubMed, and Cochrane CENTRAL were searched from 1 January 2004 to 29 July 2026. Proportions were pooled using random-intercept binomial-normal generalised linear mixed models. PROSPERO: CRD420261464937 (registration not prospective). **Results**: Of 44 retained studies (12,754 participants), 31 independent studies (11,310 participants) formed the primary pool. Pooled PDR was 53.2% (95% CI 46.1–60.1; prediction interval 19–85%), a descriptive average, not a performance target. Exploratory PDR was 81.2% after positive FIT (k = 4), 49.4% after incomplete colonoscopy (k = 4), and 39.9% in screening without prior FIT enrichment (k = 3); 14 studies had mixed pathways. Pooled detection was 32.7% for polyps ≥ 6 mm (prediction interval 8–74%) and 17.7% for polyps ≥ 10 mm (4–51%). **Conclusions**: CCE PDR is a visual yield measure, not adenoma detection or diagnostic accuracy. A global CCE PDR is not a portable quality threshold; referral-pathway estimates are descriptive and hypothesis-generating. Future quality metrics require standardised pathways, denominators, and linkage to downstream clinical outcomes.

## 1. Introduction

Colorectal cancer is the second leading cause of cancer-related death worldwide, accounting for more than 1.9 million diagnoses and approximately 900,000 deaths in 2022 [1]. Optical colonoscopy remains the reference standard for detecting and removing colorectal neoplasia. However, rising demand for lower gastrointestinal investigation, together with persistent workforce and capacity constraints, has intensified interest in less-invasive complementary tests [2]. Colon capsule endoscopy (CCE) provides a swallowed, wireless examination of the colonic mucosa. Current ESGE–ESGAR guidance supports its use after incomplete colonoscopy and in selected patients with non-alarm symptoms or positive faecal testing when colonoscopy is incomplete or not feasible [3].

In optical colonoscopy, adenoma detection rate (ADR), defined as the proportion of eligible examinations in which at least one histologically confirmed conventional adenoma is identified, is a principal quality indicator. Higher ADR is associated with lower risks of post-colonoscopy colorectal cancer and related mortality. The 2024 ASGE and ACG guidance recommends a minimum ADR of 35% for patients aged 45 years or older undergoing screening, surveillance, or diagnostic colonoscopy, with thresholds of 40% in men and 30% in women. For colonoscopy after a positive faecal immunochemical test, the recommended thresholds are 50% overall, 55% in men, and 45% in women [4]. These thresholds derive from US screening, surveillance, and diagnostic populations and are presented only as context; they cannot be transferred directly to CCE or assumed to apply unchanged across different healthcare systems. CCE PDR, however, is a visual measure without index histology and is not a CCE equivalent of ADR.

CCE, by contrast, has no validated PDR benchmark against which service quality can be judged. Reported per-patient yields range from below 20% to above 80%, reflecting differences in referral pathway, underlying polyp prevalence, bowel preparation, examination completion, reader practice, and capsule generation (Table 1). A 2024 meta-analysis already pooled CCE PDR against colonoscopy PDR, but did not stratify single-arm CCE yield by referral pathway [5]. Other syntheses, including an umbrella meta-review from this group and an updated CCE2 meta-analysis, have focused on sensitivity and specificity against a reference standard rather than on observed single-arm yield [6,7]. These are different estimands. Pooling clinically heterogeneous populations can conflate case mix with test performance: FIT-positive screening enriches for colorectal neoplasia, whereas symptomatic, screening, post-incomplete-colonoscopy, surveillance, and mixed pathways carry different pre-test probabilities. A useful description of CCE yield must therefore retain referral-pathway context and must not be mistaken for diagnostic accuracy or a quality threshold.

We therefore conducted a systematic review and meta-analysis to estimate per-patient any-polyp CCE detection yield overall and across referral pathways, to quantify size-stratified yield, and to explore study-level factors associated with between-study variation. Outcome definitions, denominator conventions, source data, and potential cohort overlap were examined to minimise extraction error. Our aim was descriptive: to map the distribution of observed CCE polyp yield and to identify what the existing evidence does—and does not—support about future quality-assurance research.

## 2. Methods

This review was reported according to the Preferred Reporting Items for Systematic Reviews and Meta-Analyses (PRISMA) 2020 statement (completed PRISMA 2020 checklist provided in the Supplementary Material) [52]. The review was submitted to PROSPERO on 29 July 2026 after preliminary searching and screening had begun; registration was therefore not prospective (CRD420261464937). The definitive database search was rerun in full on 29 July 2026. The working protocol and subsequent source-verification amendments are documented in the Supplementary and are available from the corresponding author on reasonable request. The primary outcome was the per-patient any-polyp detection rate. Secondary outcomes were detection of polyps measuring at least 6 mm or at least 10 mm, referral-pathway-specific detection rates, and exploratory associations with capsule generation, publication year, denominator convention, bowel-preparation adequacy, completion, region, age, and sex. Because registration was not prospective, all moderator and subgroup findings are interpreted as exploratory. Stratification by clinical indication was prespecified, with a cohort assigned to a single indication when at least 80% of participants shared it; the review timeline and protocol deviations are reported in Supplementary Tables S8 and S9.

### 2.1. Eligibility Criteria

We included prospective and retrospective cohort studies, cross-sectional studies, and the CCE arm of randomised or comparative studies that reported an extractable per-patient any-polyp and/or size-threshold (≥6 mm or ≥10 mm) detection rate. Eligible studies included adults undergoing a dedicated colon capsule or a panenteric capsule when colonic results were separately extractable. We applied no language restrictions, and translated non-English reports where necessary. We excluded reviews, editorials, protocols, conference abstracts, case reports, studies with fewer than 10 participants, technical or artificial-intelligence algorithm-validation studies, animal or phantom studies, gastric or small-bowel-only capsule studies, and cohorts restricted to patients with known polyps or advanced cancer. Studies reporting only per-lesion or per-frame outcomes, or comparative diagnostic accuracy without an extractable per-patient outcome, were also excluded. The minimum sample size of 10 was a pragmatic lower bound to avoid case-series-scale proportion estimates. Full eligibility criteria are provided in Supplementary Table S1.

### 2.2. Information Sources

I.I.L. searched PubMed, MEDLINE (Ovid), Embase (Ovid), and Cochrane CENTRAL from 1 January 2004 to 29 July 2026. A preliminary search was run in July 2026, and the complete search was rerun on 29 July 2026 to capture newly indexed reports. The strategy combined controlled vocabulary, including MeSH and Emtree terms, with free-text terms for colon capsule endoscopy and colorectal polyp detection. Reference lists of included studies and relevant reviews were screened, identifying seven additional reports. Full database-specific strategies are provided in Supplementary S2. No grey-literature or registry records were included in the quantitative synthesis.

### 2.3. Study Selection and Data Compilation

**Figure 1:**
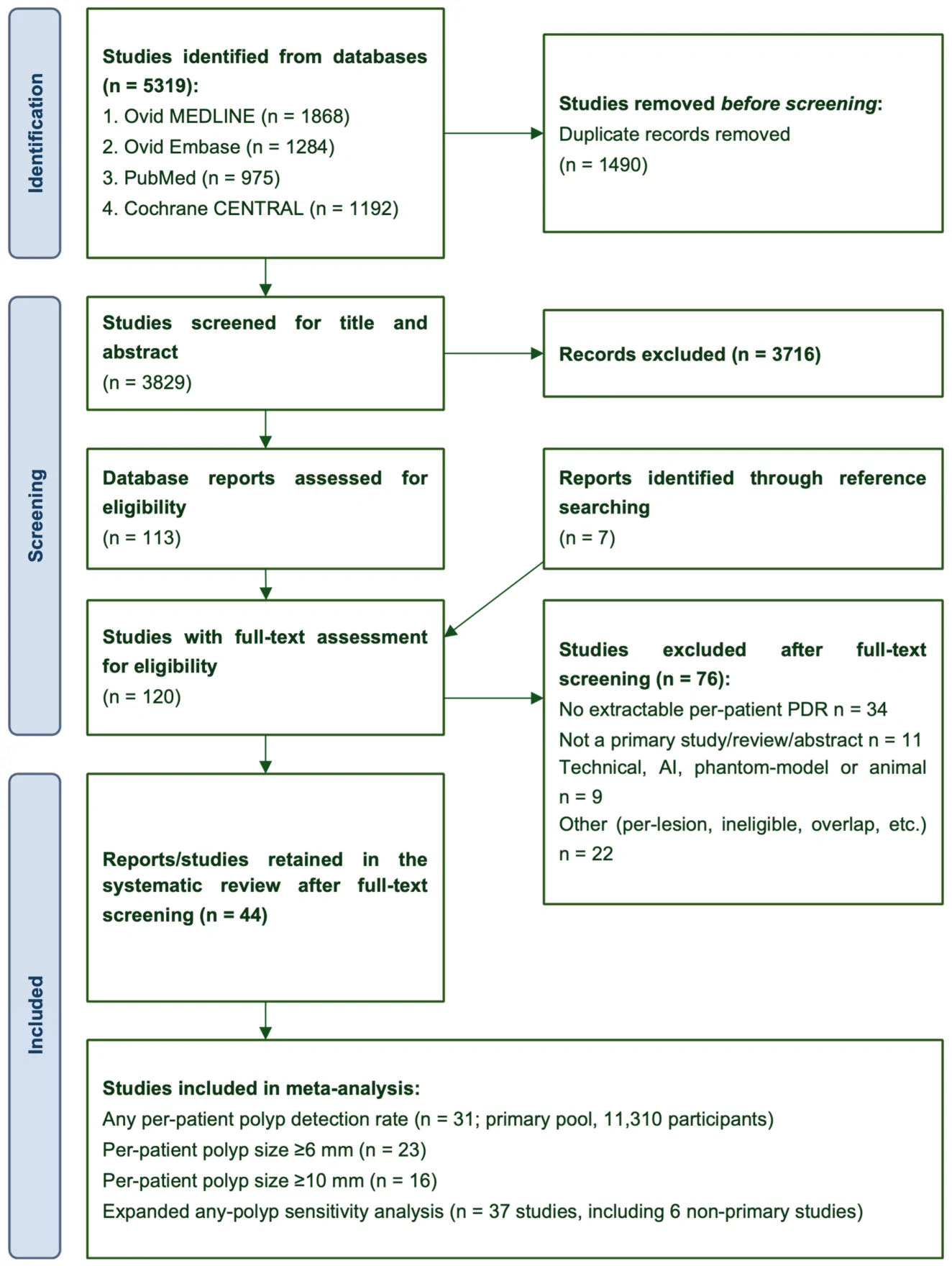
PRISMA 2020 flow diagram.

I.I.L. and G.B.V. performed title and abstract screening and full-text assessment independently using Rayyan, resolving disagreements by consensus [53] (Figure 1). I.I.L. and G.B.V. independently extracted data using a standardised form and reconciled discrepancies by consensus. Extracted characteristics included first author, publication year, country, study design, clinical setting, CCE referral pathway, capsule type or generation, enrolled and analysed sample sizes, and the denominator used for per-patient analyses. The preferred denominator was all patients who swallowed a capsule; when this was unavailable, we used the study-reported analysed, complete, or complete-and-adequate denominator and recorded the convention for sensitivity analysis and meta-regression. Outcomes were extracted as numerators and denominators for any-polyp detection and, where available, for polyps measuring at least 6 mm and at least 10 mm. We also recorded the precise outcome definition and whether each estimate was directly reported or reconstructed. For exploratory pathway grouping, FIT-positive screening denoted cohorts recruited after a positive FIT within a screening programme; screening without a preceding positive FIT included average-risk, familial-risk, or other screening cohorts without prior FIT enrichment; symptomatic denoted symptom-driven referral; post-incomplete colonoscopy required a documented previous incomplete optical colonoscopy; post-polypectomy surveillance required explicit surveillance after prior polypectomy/adenoma; colonoscopy declined denoted cohorts that had declined colonoscopy; other denoted single-indication cohorts outside these categories (anticipated difficult colonoscopy or post-diverticulitis follow-up); and mixed/unclassifiable included cohorts in which no indication reached the prespecified 80% threshold. Categories were assigned with the registered rule. The 80% threshold was author-defined; Cochrane guidance supports prespecifying how partly eligible populations are handled but does not prescribe this cut-off [54]. Results were similar with thresholds of 70–90% (Supplementary Table S3). The all-swallowed-capsule denominator was chosen as a pragmatic intention-to-test service-yield denominator; complete or adequate-examination denominators were used when the preferred denominator was unavailable and examined in sensitivity analyses because restricting the primary denominator to technically successful examinations could overstate real-world yield. The target intention-to-test service yield is the proportion of all participants who swallowed a capsule in whom at least one polyp was visually reported; incomplete or inadequately prepared examinations remain in the denominator, and any reported polyp counts as a detection. The pooled estimate combines different denominator conventions and is therefore not a uniform intention-to-test estimate; complete- or adequate-examination denominators describe technically successful examinations. PDR was visually reported without index histology, and size thresholds were reader-estimated. Indication mix, bowel preparation and cleansing scale, completion, reader qualification, age, sex, and, for FIT-positive cohorts, FIT threshold and programme were extracted (Supplementary Tables S11 and S12).

### 2.4. Risk of Bias

I.I.L. and G.B.V. assessed risk of bias independently using the nine-item JBI critical appraisal checklist for prevalence studies [55], resolving disagreements by consensus. Forty-eight candidate reports underwent item-level appraisal before overlap resolution; four overlapping or auxiliary reports were subsequently excluded, leaving 44 reports/studies in the retained corpus. The JBI checklist does not define low/moderate/high overall categories; by convention, JBI leaves this to reviewers [55]. Author-defined JBI score thresholds were used: an item scored no or unclear was counted as not met, and overall risk was low with 0–1, moderate with 2–3, and high with four or more unmet items. This mapping reproduced 18 of the 22 overall ratings on which both reviewers had independently agreed; agreed ratings stood, and disagreements were reconciled item by item against the source report (Supplementary S4.1). Certainty was assessed using a GRADE-informed framework adapted from guidance for prognostic event rates, starting at high certainty and rating down for risk of bias, inconsistency, indirectness, imprecision, and publication bias (Supplementary S8 and Table S6).

### 2.5. Statistical Analysis

The primary analysis used a random-intercept binomial-normal generalised linear mixed model (GLMM) with a logit link [56], fitted with rma.glmm (measure = “PLO”) in the R package metafor [57,58]. For proportion models, rma.glmm uses maximum-likelihood estimation with adaptive Gauss–Hermite quadrature; the metafor default of seven quadrature points was retained. This likelihood-based model accommodates zero-event cells without a continuity correction. The GLMM was preferred to the Freeman–Tukey double-arcsine transformation [59] because study sizes ranged from 10 to 4878 participants and double-arcsine back-transformation can be unstable when sample sizes vary markedly. Pooled proportions, 95% CIs, and 95% prediction intervals were estimated on the logit scale and back-transformed. A Freeman–Tukey random-effects model with restricted maximum-likelihood estimation was fitted as a sensitivity analysis [59]. Heterogeneity was characterised primarily using τ^2^ and prediction intervals; I^2^ was also reported. Prediction intervals and I^2^ were taken from meta::metaprop (GLMM), as in the forest plots. Formal funnel-plot asymmetry testing was not used because substantial clinical heterogeneity and the conditions required for meaningful asymmetry testing were not met [60]; reporting bias was considered qualitatively in relation to search coverage, publication type, study size, and outcome availability. Exploratory GLMM meta-regression examined referral pathway, capsule generation, publication year, denominator type, adequate preparation, completion, region, age, and sex; variance associated with referral pathway was summarised as the relative reduction in τ^2^ (pseudo-R^2^). Post hoc analyses comprised capsule generation adjusted for publication year and within the mixed stratum, age adjusted for pathway, pooling by denominator convention, application of the score thresholds to every study, single-indication thresholds of 70% to 90%, comparison of studies with <100 and ≥100 participants, and a descriptive regression of logit PDR on 1/√*n*. Prediction intervals were reported for pooled pathway strata with at least three studies. Single-study pathway strata were reported descriptively rather than interpreted as pooled subgroup estimates. The capsule-generation moderator was restricted to studies clearly classifiable as CCE1 or CCE2; non-PillCam and unclear devices were excluded. The expanded any-polyp sensitivity analysis included 37 datasets, including six non-primary datasets, and was not interpreted as an independent-study pool. Analyses were conducted in R version 4.5.3 [61] with metafor version 4.8-0 [57] and meta version 8.5-0 [62]. Reader experience was not meta-regressed because it was too inconsistently reported.

### 2.6. Use of Generative AI Tools

During manuscript preparation, OpenAI ChatGPT (GPT-5.6 Sol; accessed on August–September 2026) was used by A.K. for editorial quality control, including language refinement, structural review, source-checking support, and internal-consistency checks. ChatGPT (OpenAI) and Claude (Anthropic) also supported consistency checking of the manuscript, reviewer responses and Supplementary Files, including numerical cross-checks. All AI-assisted outputs were independently reviewed against the source data and publications by the authors; no generative AI tool made final decisions on study eligibility, data extraction, risk-of-bias assessment, statistical analysis, or interpretation. The authors take full responsibility for the final content.

## 3. Results

### 3.1. Literature Search and Study Selection

The definitive search on 29 July 2026 identified 5319 database records. After removal of 1490 duplicates, 3829 records were screened, and 3716 were excluded. Full texts were assessed for 113 database reports; reference searching identified seven additional reports, bringing the total to 120 full-text reports assessed. Seventy-six reports were excluded, leaving 44 retained reports/studies [8,9,10,11,12,13,14,15,16,17,18,19,20,21,22,23,24,25,26,27,28,29,30,31,32,33,34,35,36,37,38,39,40,41,42,43,44,45,46,47,48,49,50,51]. Figure 1 shows the reasons for exclusion. Before the final eligibility and overlap decision, 48 candidate reports underwent risk-of-bias appraisal; four overlapping or auxiliary reports were subsequently excluded. The 44 study-level rows in Table 1 sum to 12,754 participants. Thirty-seven studies contain an extractable per-patient any-polyp numerator (12,013 participants); their disposition is described in Section 3.2 and Supplementary S6.1.

### 3.2. Overall Results

**Figure 2:**
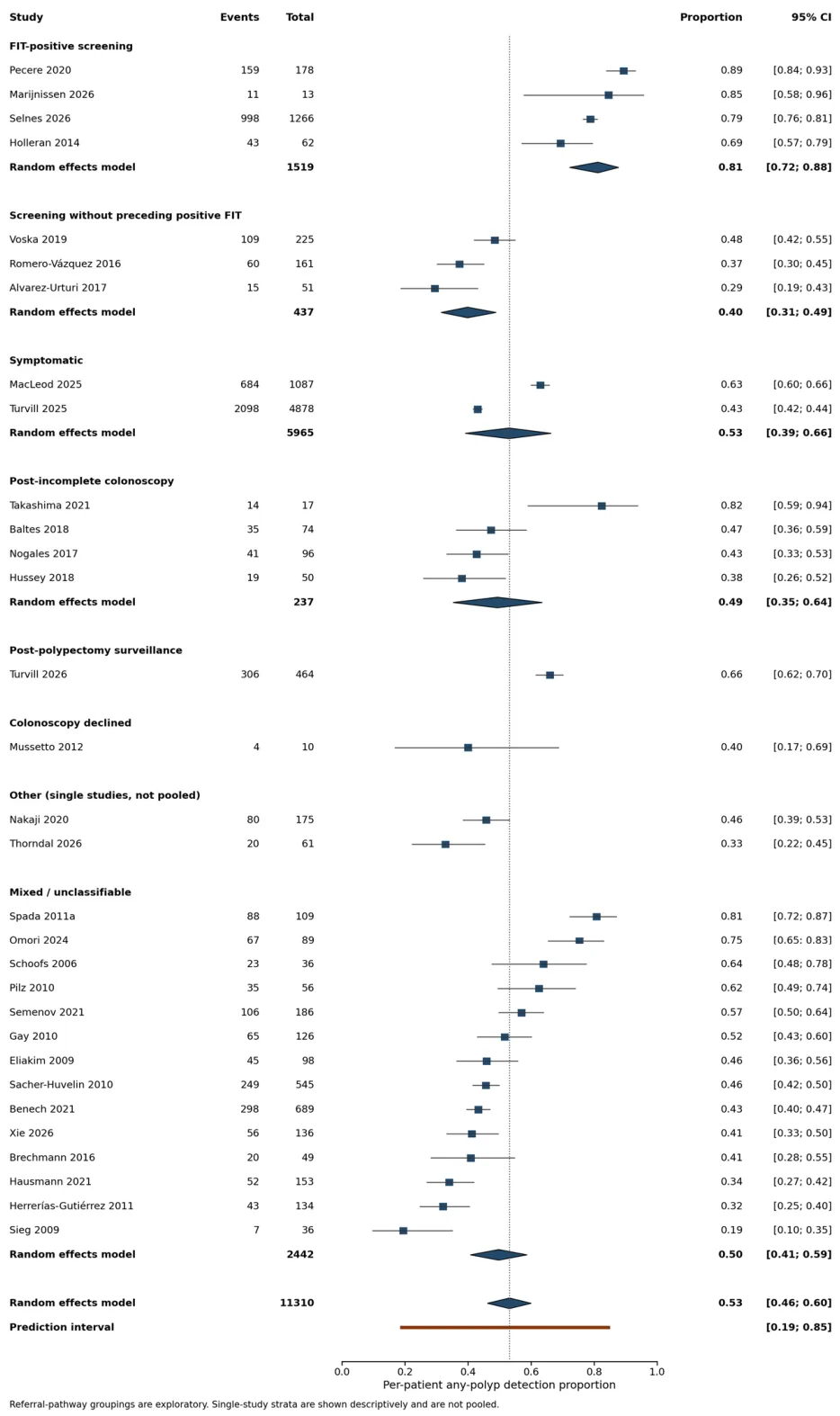
Forest plot of the per-patient any-polyp detection rate (visually reported, without histology) of colon capsule endoscopy by exploratory referral pathway (primary pool, k = 31; registered ≥80% rule). These groupings are exploratory and descriptive, not confirmatory subgroup analyses; 14 of 31 studies could not be assigned to one pathway. Pooled GLMM estimates are shown only for groups of two or more studies; single studies are not pooled. Substantial heterogeneity remains within groups (I^2^ 66–99%). Studies are labelled by first author and publication year: Schoofs 2006 [8]; Eliakim 2009 [9]; Sieg 2009 [10]; Gay 2010 [11]; Pilz 2010 [12]; Sacher-Huvelin 2010 [13]; Herrerías-Gutiérrez 2011 [14]; Spada 2011a [15]; Mussetto 2012 [17]; Holleran 2014 [19]; Brechmann 2016 [22]; Romero-Vázquez 2016 [24]; Alvarez-Urturi 2017 [25]; Nogales 2017 [26]; Baltes 2018 [27]; Hussey 2018 [28]; Voska 2019 [30]; Nakaji 2020 [33]; Pecere 2020 [34]; Benech 2021 [35]; Hausmann 2021 [36]; Semenov 2021 [37]; Takashima 2021 [38]; Omori 2024 [41]; MacLeod 2025 [43]; Turvill 2025 [45]; Marijnissen 2026 [47]; Selnes 2026 [48]; Thorndal 2026 [49]; Turvill 2026 [50]; Xie 2026 [51].

Thirty-seven studies reported an extractable any-polyp outcome (12,013 participants). Six non-primary datasets were excluded from the primary model because of non-uniform outcome definitions, overlapping cohorts, or a reconstructed numerator [18,32,39,42,44,46], leaving 31 independent studies and 11,310 participants (Supplementary S6.1 and Table S10). The pooled per-patient PDR was 53.2% (95% CI 46.1–60.1%), an average across studies rather than an expected value for any individual service (see Figure 2). Between-study variation was substantial, with a 95% prediction interval of 19–85% and τ^2^ = 0.57. The crude aggregate proportion was 51.7%, and a Freeman–Tukey sensitivity model yielded 52.8%. I^2^ was approximately 96%. Given the extreme clinical heterogeneity, no formal funnel-asymmetry test was interpreted as evidence for or against reporting bias.

### 3.3. Size-Stratified Detection

In analyses restricted to provenance-verified, non-overlapping cohorts, the pooled per-patient detection rate was 32.7% (95% CI 25.4–41.0; 95% prediction interval 8–74%) for polyps measuring at least 6 mm across 23 studies and 17.7% (12.7–24.1; 95% prediction interval 4–51%) for polyps measuring at least 10 mm across 16 studies. Estimates were stable in leave-one-out analyses, ranging from 31.1% to 34.7% and from 16.3% to 19.2%, respectively. Both thresholds are reader-estimated sizes at capsule reading, not histological measurements.

### 3.4. Risk of Bias Assessment

Risk-of-bias assessments were completed independently by two reviewers and disagreements were resolved by consensus; no unreconciled ratings remained. Forty-eight candidate reports were appraised before final overlap resolution. Among the 44 retained reports/studies, nine (20%) were judged low-risk, 20 (45%) moderate-risk, and 15 (34%) high-risk using the author-defined overall ratings summarised in Supplementary S4.1 and Figure S1; applying the score thresholds to every report reclassified five of 44 by one category. Principal concerns were non-consecutive or poorly described sampling and incomplete CCE examinations or follow-up. Referral pathways were frequently unclear or insufficiently disaggregated, limiting pathway-specific interpretation.

### 3.5. Sensitivity Analyses and Meta-Regression

The pooled estimate was stable across sensitivity analyses: 51.7% in the expanded 37-dataset sensitivity analysis, which included six non-primary datasets and was not an independent-study pool; 51.3% after excluding the five reconstructed-count studies present in the primary pool; and 55.8% after excluding high-risk studies, or 58.0% when the score thresholds were applied to every study (Supplementary Table S5). Leave-one-out estimates ranged from 51.4% to 54.3%.

Referral pathway was associated with PDR in exploratory meta-regression (omnibus QM = 28.2, df = 7, *p* < 0.001; pseudo-R^2^ 55%), although residual heterogeneity remained high (I^2^ 92%). Among studies clearly classifiable as CCE1 or CCE2, CCE2 showed higher PDR in unadjusted analysis (OR 2.17, 95% CI 1.19–3.95; *p* = 0.012), but the association attenuated after adjustment for referral pathway (OR 1.67, 95% CI 0.95–2.92; *p* = 0.073) and became imprecise after adjustment for publication year (OR 2.16, 0.95–4.92; *p* = 0.067). Capsule generation was collinear with pathway and era: all four FIT-positive cohorts used CCE2, and the median publication year was 2010 for CCE1 and 2021 for CCE2 (Supplementary Table S7). Within the mixed stratum (seven CCE1 and six CCE2 studies), the OR was 1.62 (0.77–3.38; *p* = 0.20). Publication year (*p* = 0.25) and denominator convention (*p* = 0.61) showed no clear association with PDR, but these nonsignificant tests do not establish equivalence or absence of effect. PDR was 54.3% (95% CI 48.0–60.5; k = 23) with all-swallowed or analysable-video denominators and 49.9% (29.2–70.7; k = 7) with complete or adequate-examination denominators. Adequate preparation (reported in 26 studies; median 78%, range 38–96%; validated scale in 12) and completion (31 studies; median 81%, range 57–95%) were not associated with PDR (OR 1.08 per 10 percentage points, 95% CI 0.89–1.31; *p* = 0.46 and OR 0.96, 0.74–1.24; *p* = 0.75, respectively; Supplementary Tables S4 and S12), although study-level analyses cannot exclude a patient-level effect. Region showed no clear association (Asia/Middle East, k = 5, versus Europe, k = 26; OR 1.24, 0.57–2.68; *p* = 0.59). Older cohorts had a higher PDR (OR 1.87 per 10 years, 95% CI 1.15–3.04; *p* = 0.012; k = 30), but this attenuated after adjustment for referral pathway (OR 1.48, 0.98–2.23; *p* = 0.059); the proportion of men was not associated with PDR (OR 1.10 per 10 percentage points, 0.89–1.37; *p* = 0.37; k = 29). Reader qualification was only quantified in five studies, described qualitatively in 18, and not stated in eight. Studies with <100 participants had a PDR of 51.1% (95% CI 41.0–61.2; k = 15) versus 54.8% (45.2–64.0; k = 16) in larger studies.

### 3.6. Exploratory Subgroup Analysis by Referral Pathway

Pathways were assigned with the prespecified ≥80% single-indication rule. Accordingly, the ScotCap registry [43] (95.9% symptomatic) was classed as symptomatic, Romero-Vázquez 2016 [24] (81.2% screening) as screening, Nakaji 2020 [33] (90.9% anticipated difficult colonoscopy) and Thorndal 2026 [49] (post-diverticulitis follow-up) as other, and Mussetto 2012 [17] (FOBT-positive patients who had declined colonoscopy) as colonoscopy declined. Familial-risk and other screening cohorts without a preceding positive FIT were grouped as screening without prior FIT enrichment. In the primary pool, PDR was 81.2% (95% CI 72.2–87.8; 95% PI 47–96%; k = 4) in FIT-positive screening, 49.4% (35.2–63.6; PI 15–84%; k = 4) after documented incomplete colonoscopy, 39.9% (31.5–49.0; PI 15–72%; k = 3) in screening without a preceding positive FIT, 53.0% (39.1–66.4; k = 2) in symptomatic cohorts, and 49.7% (40.7–58.8; PI 18–81%; k = 14) in mixed/unclassifiable cohorts, with substantial heterogeneity within strata (I^2^ 66–99%; Table 2). Single studies reported 65.9% in post-polypectomy surveillance [50], 40.0% after declined colonoscopy [17], 45.7% with anticipated difficult colonoscopy [33], and 32.8% after diverticulitis [49]; these were not pooled. FIT-positive cohorts differed in FIT threshold (20–47 µg Hb/g where reported), route to CCE, and denominator, and two [34,48] used complete-examination denominators (Supplementary Table S11). These categories remain exploratory and should not be used as pathway-specific reference values. Retaining the five reclassified cohorts in their source-level categories gave a similar pattern (Supplementary Table S3).

## 4. Discussion

Prior syntheses have addressed overall CCE PDR versus colonoscopy PDR [5] and diagnostic accuracy against colonoscopy or CT colonography [6,7,63]. To our knowledge, the present study is the first single-arm meta-analysis to disaggregate per-patient CCE polyp yield by referral pathway while also examining denominator convention and capsule generation. The principal finding is not a universal benchmark: CCE identified at least one visually reported polyp in approximately half of patients overall, but the 19–85% prediction interval shows that a new setting should not be expected to reproduce the pooled mean. Referral pathway was associated with about half of the between-study variance, most visibly through the high yield in FIT-positive screening, but 14 of 31 primary studies remained mixed, post-polypectomy surveillance was a single study, and heterogeneity persisted within strata; neither the pooled 53.2% nor the pathway estimates should be used as performance standards. The association between CCE2 and higher yield attenuated after adjustment for pathway; adjustment for publication year left the point estimate essentially unchanged but widened its confidence interval. CCE2 is entangled with later publication and enriched pathways. To separate device from population, we would need within-study comparisons or individual patient data, which were unavailable. Older cohorts similarly had higher yield, but this also attenuated after adjustment for pathway.

Unlike optical colonoscopy, CCE has no validated PDR threshold. In colonoscopy, ADR is a validated quality indicator, while PDR is an auditable but less specific surrogate that correlates with ADR [64,65]. CCE PDR is more limited: it records visually reported polyps without routine index histology and is jointly influenced by population selection, completion, preparation, reader practice, denominator convention, and false-positive calls. It therefore includes hyperplastic and other non-neoplastic polyps and cannot be compared numerically with colonoscopic ADR. The optical-colonoscopy relationship between PDR and ADR therefore cannot simply be imported into CCE. Paired CCE-colonoscopy studies compare yield and diagnostic accuracy but have not established a stable conversion from CCE PDR to downstream colonoscopic ADR [66]. For quality assurance, harder outcome-based measures are also emerging: an international 2026 consensus defined post-CCE colorectal cancer (pCCECRC) as a candidate service- and test-level quality metric, while emphasising the need for real-world validation [67]. PDR should therefore be considered a descriptive yield measure unless and until it is prospectively linked to clinically meaningful outcomes.

**Figure 3:**
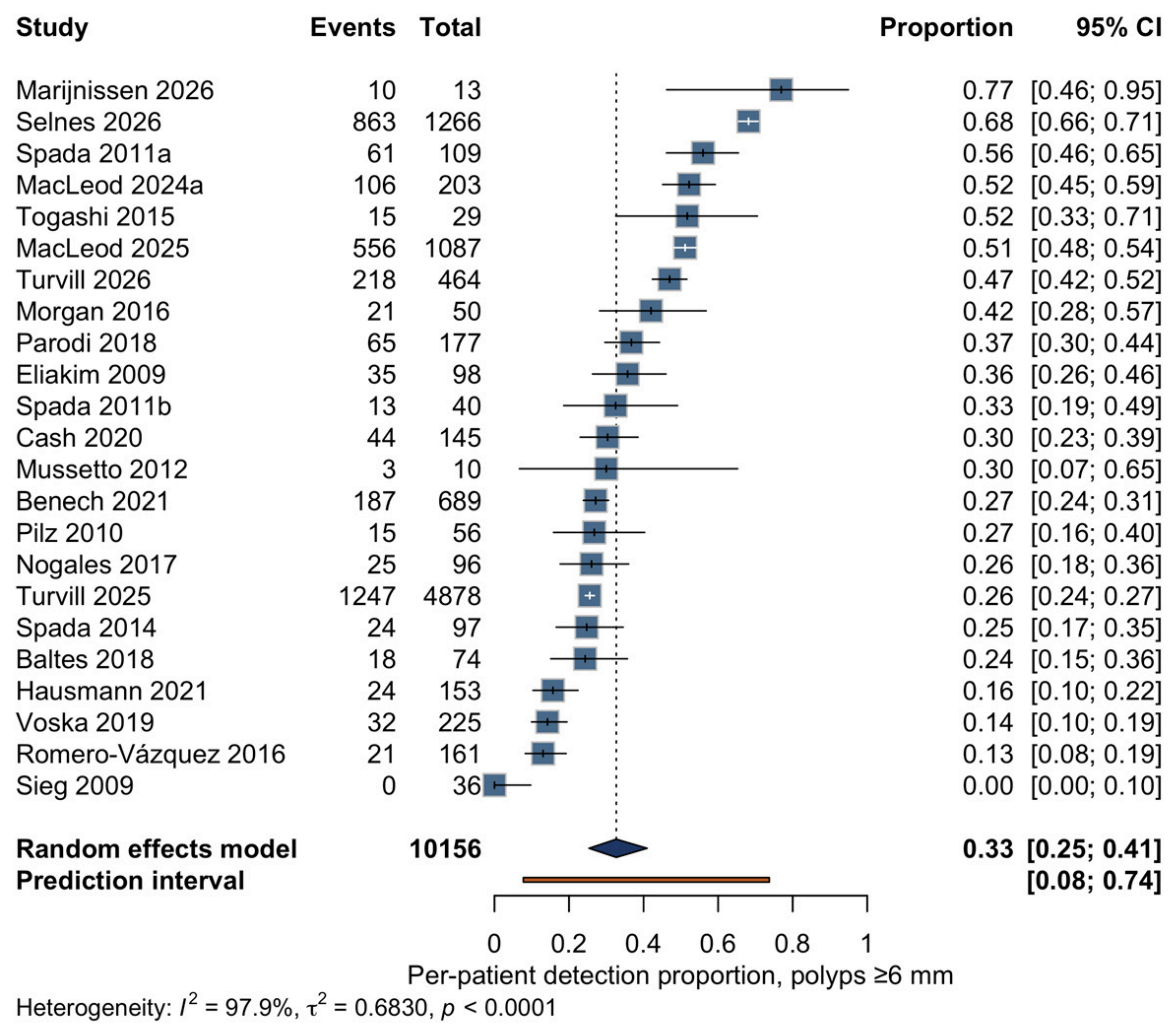
Forest plot of the pooled per-patient CCE detection rate for polyps ≥ 6 mm (k = 23). Studies are labelled by first author and publication year: Eliakim 2009 [9]; Sieg 2009 [10]; Pilz 2010 [12]; Spada 2011a [15]; Spada 2011b [16]; Mussetto 2012 [17]; Spada 2014 [20]; Togashi 2015 [21]; Morgan 2016 [23]; Romero-Vázquez 2016 [24]; Nogales 2017 [26]; Baltes 2018 [27]; Parodi 2018 [29]; Voska 2019 [30]; Cash 2020 [31]; Benech 2021 [35]; Hausmann 2021 [36]; MacLeod 2024a [40]; MacLeod 2025 [43]; Turvill 2025 [45]; Marijnissen 2026 [47]; Selnes 2026 [48]; Turvill 2026 [50].

Two considerations are important when interpreting these estimates. First, a higher PDR may increase referral for therapeutic colonoscopy and should not automatically be interpreted as better- or worse-quality. Second, PDR may change as referral pathways, capsule technology, preparation, reading practice, and artificial-intelligence-assisted analysis evolve [68,69]. Detection rates for larger lesions were 32.7% for polyps measuring at least 6 mm (95% PI 8–74%; Figure 3) and 17.7% for polyps measuring at least 10 mm (95% PI 4–51%; Supplementary Figure S2). These outcomes may be more relevant to downstream management than any-polyp yield, but they remain reader-estimated visual size thresholds rather than histological endpoints. ESGE–ESGAR guidance recommends referral for endoscopic polypectomy when CCE identifies at least one polyp ≥ 6 mm [3], making the ≥6 mm yield relevant to downstream colonoscopy demand. AI-assisted reading may also alter observed yield: in the multicentre CESCAIL analysis, per-patient polyp diagnostic yield was 70.6% with AI-assisted reading versus 63.6% with standard reading, while mean reading time fell from 47.3 to 8.7 min [70]. FIT-positive yield also depends on FIT threshold and route to CCE, so the pooled 81.2% should not be transferred between programmes. Any future reference framework should therefore specify device generation, preparation, completion criteria, denominator, and reading workflow.

Detection yield should not be interpreted as negative predictive performance. The present analysis estimates how often lesions were reported within each study population, not the proportion of clinically significant lesions missed by CCE. Residual miss rates and the safety of using CCE to avoid colonoscopy require paired reference-standard evidence. Recent syntheses of CCE diagnostic accuracy [6,7], procedural success [69], and health technology/economic evidence [71] address adjacent but distinct questions. The contribution of the present analysis is to show that observed yield itself is highly context-dependent and that a crude global PDR is unsuitable for service benchmarking. Future studies should enrol clearly defined single-pathway cohorts, prespecify FIT thresholds or symptom criteria, standardise preparation/completion and denominator definitions, and link CCE findings to colonoscopic histology, advanced neoplasia, repeat investigation, and pCCECRC rather than repeat PDR alone.

This study has several limitations. Heterogeneity remained extreme, and pooled means must be interpreted alongside prediction intervals. Studies did not use a single denominator convention: although all swallowed capsules were preferred, some estimates were based on analysed, complete, or complete-and-adequate examinations, and estimates by denominator convention differed by about four percentage points (54.3% versus 49.9%; only seven studies in the latter group). Potential cohort overlap was addressed, and the disposition of all 37 extractable datasets, including six non-primary datasets used only in the expanded sensitivity analysis, is documented in Supplementary S6.1. Counts were reconstructed in seven retained reports, five of which were present in the primary pool; excluding those five did not materially change the estimate. Many publications did not report referral pathways in sufficient detail for narrow clinical categories, so 14 of 31 primary studies remained mixed and only one study was assigned to post-polypectomy surveillance. Preparation adequacy and completion were inconsistently defined and analysable only at the study level; reader experience could not be analysed. The review excluded conference abstracts, and unpublished small low-yield studies could bias the pooled PDR upwards. A descriptive regression of logit PDR on 1/√n (*p* = 0.89) and a comparison by study size were uninformative but have little power under this heterogeneity, so reporting bias cannot be excluded. We did not rate certainty down for publication bias, because the large registries and service evaluations included are likely to be published regardless of their results. Even so, missing studies cannot be ruled out. Finally, the protocol registration was not prospective, and the moderator analyses should be regarded as exploratory (Supplementary Table S9). Certainty was low for any-polyp yield and very low for size-stratified and referral-pathway estimates (Supplementary Table S6).

## 5. Conclusions

Colon capsule endoscopy identified at least one visually reported polyp in approximately half of patients overall, but this pooled value is not a portable quality benchmark. CCE PDR is a visual yield measure, not adenoma detection or diagnostic accuracy. The wide prediction interval and source-level differences in referral pathway, device generation, preparation, completion, reading workflow, and denominator limit direct comparison between services. Referral-pathway estimates are descriptive and hypothesis-generating, and the current evidence does not justify formal PDR thresholds. Prospective single-pathway studies with standardised reporting and linkage to colonoscopic histology, advanced neoplasia, downstream intervention, and pCCECRC are required before CCE performance standards can be established.

## Figures and Tables

**Table 1:** Study characteristics.

Study	Country	Design	Referral Pathway	Capsule	Patients (N)	Any-Polyp	≥6 mm	≥10 mm	RoB
Schoofs et al., 2006 [8]	Belgium	Prospective	Mixed	CCE1	36	23/36 (64%)	—	—	moderate
Eliakim et al., 2009 [9]	Israel	Prospective	Mixed	CCE2	98	45/98 (46%)	35/98 (36%)	17/98 (17%)	moderate
Sieg et al., 2009 [10]	Germany	Prospective	Mixed	CCE1	36	7/36 (19%)	0/36 (0%)	—	high
Gay et al., 2010 [11]	France	Prospective	Mixed	CCE1	126	65/126 (52%)	—	—	moderate
Pilz et al., 2010 [12]	Switzerland	Prospective	Mixed	CCE1	56	35/56 (62%)	15/56 (27%)	—	moderate
Sacher-Huvelin et al., 2010 [13]	France	Prospective	Mixed	CCE1	545	249/545 (46%)	—	—	low
Herrerías-Gutiérrez et al., 2011 [14]	Spain	Prospective	Mixed	CCE1	134	43/134 (32%)	—	—	high
Spada et al., 2011a [15]	Italy	Prospective	Mixed	CCE2	109	88/109 (81%)	61/109 (56%)	32/109 (29%)	moderate
Spada et al., 2011b [16]	Italy	RCT arm	Mixed	CCE1	40	—	13/40 (32%)	—	high
Mussetto et al., 2012 [17]	Italy	Prospective	Colonoscopy declined (FOBT-positive)	CCE1	10	4/10 (40%)	3/10 (30%)	1/10 (10%)	high
Pioche et al., 2012 [18]	France	Prospective	Mixed	CCE1	100	26/100 (26%)	—	—	high
Holleran et al., 2014 [19]	Ireland	Prospective	FIT+ screen	CCE2	62	43/62 (69%)	—	—	moderate
Spada et al., 2014 [20]	Italy	Prospective	Post-incomplete	CCE2	97	—	24/97 (25%)	5/97 (5%)	moderate
Togashi et al., 2015 [21]	Japan	Prospective	Post-incomplete	CCE2	29	—	15/29 (52%)	8/29 (28%)	high
Brechmann et al., 2016 [22]	Germany	Prospective	Mixed	CCE1	49	20/49 (41%)	—	1/49 (2%)	high
Morgan et al., 2016 [23]	USA	Prospective	Mixed	CCE2	50	—	21/50 (42%)	10/50 (20%)	moderate
Romero-Vázquez et al., 2016 [24]	Spain	Prospective	Screening (81% of cohort)	Unclear	161	60/161 (37%)	21/161 (13%)	—	moderate
Alvarez-Urturi et al., 2017 [25]	Spain	Prospective	Familial-risk screening	CCE1	51	15/51 (29%)	—	—	moderate
Nogales et al., 2017 [26]	Spain	Prospective	Post-incomplete	CCE2	96	41/96 (43%)	25/96 (26%)	—	moderate
Baltes et al., 2018 [27]	Germany	Prospective	Post-incomplete	CCE2	74	35/74 (47%)	18/74 (24%)	—	low
Hussey et al., 2018 [28]	Ireland	Prospective	Post-incomplete	Unclear	50	19/50 (38%)	—	—	high
Parodi et al., 2018 [29]	Italy and France	Prospective	Familial-risk screening	CCE2	177	—	65/177 (37%)	32/177 (18%)	low
Voska et al., 2019 [30]	Czech Republic	Prospective	Average-risk screening	CCE2	225	109/225 (48%)	32/225 (14%)	16/225 (7%)	low
Cash et al., 2020 [31]	USA	RCT arm	Average-risk screening	CCE2	145	—	44/145 (30%)	19/145 (13%)	low
González-Suárez et al., 2020 [32]	Spain	RCT arm	FIT+ screen	CCE2	145	118/145 (81%)	—	—	low
Nakaji et al., 2020 [33]	Japan	Retrospective	Other (anticipated difficult colonoscopy, 91%)	CCE2	175	80/175 (46%)	—	—	high
Pecere et al., 2020 [34]	Italy, Spain	Prospective	FIT+ screen	CCE2	178	159/178 (89%)	—	—	moderate
Benech et al., 2021 [35]	France	Prospective	Mixed	CCE2	689	298/689 (43%)	187/689 (27%)	—	moderate
Hausmann et al., 2021 [36]	Germany	Prospective	Mixed	CCE2	153	52/153 (34%)	24/153 (16%)	—	moderate
Semenov et al., 2021 [37]	Ireland	Prospective	Mixed	CCE2	186	106/186 (57%)	—	—	moderate
Takashima et al., 2021 [38]	Japan	Prospective	Post-incomplete	CCE2	17	14/17 (82%)	—	—	high
Semenov et al., 2022 [39]	Ireland	Retrospective	Mixed	CCE2	184	96/184 (52%)	—	—	high
MacLeod et al., 2024a [40]	UK	Prospective	Symptomatic	Unclear	203	—	106/203 (52%)	49/203 (24%)	moderate
Omori et al., 2024 [41]	Japan	Prospective	Mixed	CCE2	89	67/89 (75%)	—	—	low
Benamouzig et al., 2025 [42]	France	Prospective	Symptomatic	Panenteric capsule	29	4/29 (14%)	—	—	high
MacLeod et al., 2025 [43]	United Kingdom	Prospective	Symptomatic (96% of cohort)	CCE2	1087	684/1087 (63%)	556/1087 (51%)	294/1087 (27%)	moderate
Semenov et al., 2025 [44]	Ireland	Prospective	Post-polypectomy surveillance	CCE2	126	89/126 (71%)	—	—	moderate
Turvill et al., 2025 [45]	UK	Prospective	Symptomatic	CCE2	4878	2098/4878 (43%)	1247/4878 (26%)	647/4878 (13%)	low
Agache et al., 2026 [46]	Denmark	Prospective	Mixed	CC100	119	25/119 (21%)	—	—	moderate
Marijnissen et al., 2026 [47]	The Netherlands	Prospective	FIT+ screen	CCE2	13	11/13 (85%)	10/13 (77%)	5/13 (38%)	high
Selnes et al., 2026 [48]	Denmark	RCT arm	FIT+ screen	CCE2	1266	998/1266 (79%)	863/1266 (68%)	579/1266 (46%)	moderate
Thorndal et al., 2026 [49]	Denmark	RCT arm	Other (post-diverticulitis follow-up)	Unclear	61	20/61 (33%)	—	—	high
Turvill et al., 2026 [50]	United Kingdom	Prospective	Post-polypectomy surveillance	CCE2	464	306/464 (66%)	218/464 (47%)	95/464 (20%)	high
Xie et al., 2026 [51]	China	Prospective	Mixed	CC100	136	56/136 (41%)	—	—	low

Any-polyp/≥6 mm/≥10 mm = per-patient CCE detection (numerator/denominator). “—” indicates that the outcome was not reported or could not be source-verified. Values are post-verification. RoB = author-defined overall category derived from the reconciled JBI item-level appraisal (low/moderate/high). Seven studies contribute size-threshold data only. Thirty-seven of the 44 studies have extractable any-polyp numerators; the 31-study primary pool is documented in Supplementary S6.1 and Table S10. Referral pathways follow the registered ≥80% single-indication rule, and cohorts below this threshold are shown as mixed (indication mix in Supplementary Table S12).

**Table 2:** Pooled per-patient detection rates, heterogeneity, certainty, and exploratory estimates by referral pathway.

**Outcome**	**k**	**Pooled PDR (95% CI)**	**95% PI**	**I^2^**	**GRADE Certainty**
Any-polyp: primary (de-duplicated, uniform definition, *n* ≥ 10)	31	53.2% (46.1–60.1)	19–85%	96%	Low
Expanded 37-dataset sensitivity analysis (includes six non-primary studies)	37	51.7% (44.6–58.8)	16–86%	96%	Low
Polyps ≥ 6 mm	23	32.7% (25.4–41.0)	8–74%	98%	Very low
Polyps ≥ 10 mm	16	17.7% (12.7–24.1)	4–51%	98%	Very low

**Exploratory referral-pathway strata (primary pool; registered ≥80% rule); formal pooling shown only for k ≥ 2**					
**Referral pathway**		**k**		**PDR (95% CI); 95% PI; I^2^**	
**FIT-positive screening**		4		81.2% (72.2–87.8); 47–96%; 79%	
**Mixed/unclassifiable**		14		49.7% (40.7–58.8); 18–81%; 89%	
**Post-incomplete colonoscopy**		4		49.4% (35.2–63.6); 15–84%; 66%	
**Screening without preceding positive FIT**		3		39.9% (31.5–49.0); 15–72%; 76%	
**Post-polypectomy surveillance**		1		65.9% (single study; not pooled)	
**Symptomatic**		2		53.0% (39.1–66.4); —; 99%	
**Colonoscopy declined**		1		40.0% (single study; not pooled)	
**Other: anticipated difficult colonoscopy**		1		45.7% (single study; not pooled)	
**Other: post-diverticulitis follow-up**		1		32.8% (single study; not pooled)	

## Data Availability

Study-level extraction data, pooled studies and overlap decisions, item-level risk-of-bias ratings, and the complete R analysis code are provided as Supplementary Materials (Table S13 and Code S1).

## Supplementary Materials

The following supporting information can be downloaded at: https://www.mdpi.com/article/10.3390/diagnostics16213493/s1. Supplementary R1 (Figure S1: Risk of bias—JBI critical-appraisal checklist for prevalence studies; Figure S2: Forest plot of the pooled per-patient CCE detection rate for polyps ≥10 mm; Table S1: Eligibility criteria (inclusion and exclusion); Table S2: Pooled per-patient detection rates (any-polyp, ≥6 mm, ≥10 mm), with heterogeneity; Table S3: Any-polyp detection rate by exploratory referral pathway (primary pool; registered ≥80% single-indication rule); Table S4: Meta-regression of candidate moderators (univariable unless stated); Table S5: Sensitivity analyses (any-polyp); Table S6: GRADE: certainty of evidence; Table S7: Capsule generation by referral pathway (primary pool, registered classification), with median publication year; Table S8: Timeline of protocol registration, searching, and analysis; Table S9: Deviations from the registered protocol (PROSPERO CRD420261464937); Table S10: Derivation of the analytic pools from the 44 retained studies; Table S11: FIT-positive cohorts: FIT threshold, programme, and route to CCE; Table S12: Study-level indication mix, denominator, bowel preparation, completion, reader qualification, age, and sex (primary pool)); Table S13: Study-level dataset; Code S1: R analysis script; and the PRISMA 2020 checklist. References [72,73,74,75,76] are cited in the Supplementary Materials.

## Abbreviations

ADR	Adenoma detection rate
AI	Artificial Intelligence
CCE	Colon capsule endoscopy
CCE1	PillCam COLON 1
CCE2	PillCam COLON 2
CI	Confidence interval
COVID-19	Coronavirus Disease 2019
CRC	Colorectal Cancer
CT	Computed tomography
CTC	Computed tomography colonography
ESGE	European Society of Gastrointestinal Endoscopy
FIT	Faecal Immunochemical Test
GI	Gastrointestinal
GLMM	Generalised Linear Mixed Model
GRADE	Grading of Recommendations, Assessment, Development and Evaluations
JAG	Joint Advisory Group
JBI	Joanna Briggs Institute
KPIs	Key Performance Indicators
MeSH	Medical Subject Headings
NHS	National Health Service
OR	Odds Ratio
PCE	Panenteric Capsule Endoscopy
PDR	Polyp detection rate
PI	Prediction interval
PICO	Population, Intervention, Comparison and Outcome
PRISMA	Preferred Reporting Items for Systematic Reviews and Meta-Analyses
PROSPERO	International Prospective Register of Systematic Reviews
REML	Restricted Maximum Likelihood
