# Supplementary material for: Per-Patient Polyp Detection Rate of Colon Capsule Endoscopy: A Systematic Review and Meta-Analysis with Exploratory Stratification by Referral Pathway
Source: Diagnostics (Basel). 2026 Oct 25;16(21):3493. doi: 10.3390/diagnostics16213493 (PMC13762387; doi:10.3390/diagnostics16213493)
Supplement: Supplementary file 1 [file diagnostics-16-03493-s001.zip › diagnostics-4617598-supplementary.zip!/diagnostics-4617598-supplementary.pdf]

Supplementary Materials

Article title: Per-Patient Polyp Detection Rate of Colon Capsule Endoscopy: A Systematic Review and Meta-Analysis with Exploratory Stratification by Referral Pathway

Journal: Diagnostics

Authors: Ian Io Lei; Grazia Bernui Vigo; Ramesh P. Arasaradnam; Anastasios Koulaouzidis

Corresponding author: Ian Io Lei (Brian.lei@uhcw.nhs.uk)

S1. Databases, dates, and protocol

- Databases searched: Ovid MEDLINE, Ovid Embase, PubMed, Cochrane CENTRAL.
- Date range of records: 01 January 2004 – 29 July 2026.
- Search executed: first run July 2026; re-run in full on 29 July 2026, which is the definitive search date used throughout.
- PROSPERO record was submitted on 29 July 2026; registration number: CRD420261464937; registration approved on 19 August 2026 (timeline in Table S8). Preliminary searching and screening had already begun, so registration was not prospective. The full protocol was finalised on 26 July 2026, and version 3.0 (dated 29 July 2026) is the version uploaded with the PROSPERO record; post-registration source-verification refinements are described in S6–S7. The protocol is available from the corresponding author on reasonable request.
- Reporting standard: PRISMA 2020.
- Records identified: Ovid MEDLINE 1,868 · Ovid Embase 1,284 · PubMed 975 · Cochrane CENTRAL 1,192 — total 5,319.

S2. Full search strategies

Topic: Colon capsule endoscopy for detection of colorectal polyps, adenomas, lesions, and colorectal neoplasia/cancer. Two concepts combined with AND: (1) colon capsule endoscopy; (2) colorectal polyp/neoplasia.

S2.1 PubMed

```
(
  "colon capsule endoscopy"[tiab] OR "colonic capsule endoscopy"[tiab]
  OR "colon capsule examination"[tiab] OR "colonic capsule examination"[tiab]
  OR "capsule colonoscopy"[tiab] OR "colon capsule"[tiab] OR "colonic capsule"[tiab]
  OR "colon video capsule"[tiab] OR "colonic video capsule"[tiab]
  OR "PillCam COLON"[tiab] OR "PillCam Colon 2"[tiab] OR "PillCam COLON2"[tiab]
```

OR "second-generation colon capsule"[tiab] OR "second generation colon capsule"[tiab]
OR "CCE-2"[tiab] OR CCE2[tiab]
OR ((capsule endoscop*[tiab] OR video capsule*[tiab]) AND (colon*[tiab] OR colorect*[tiab]))
)
AND
(
"Colonic Polyps"[Mesh] OR "Adenoma"[Mesh] OR "Colorectal Neoplasms"[Mesh]
OR polyp*[tiab] OR polypoid[tiab] OR polypoidal[tiab] OR adenoma*[tiab]
OR neoplas*[tiab] OR carcinoma*[tiab] OR cancer*[tiab] OR tumor*[tiab] OR tumour*[tiab] OR lesion*[tiab]
OR "colorectal cancer"[tiab] OR "colon cancer"[tiab] OR "rectal cancer"[tiab]
OR "advanced adenoma"[tiab] OR "advanced neoplasia"[tiab]
OR "serrated lesion"[tiab] OR "serrated polyp"[tiab]
OR "sessile serrated lesion"[tiab] OR "sessile serrated polyp"[tiab]
)
AND ("2004/01/01"[dp] : "2026/07/29"[dp])

S2.2 Ovid Embase

1. (colon capsule endoscop* or colonic capsule endoscop* or colon capsule examination* or colonic capsule examination* or capsule colonoscop* or colon capsule* or colonic capsule* or colon video capsule* or colonic video capsule*).ti,ab,kw.
2. ((capsule endoscop* or video capsule*) adj3 (colon* or colorect*)).ti,ab,kw.
3. (capsule* adj3 (colonoscop* or colon* or colorect*)).ti,ab,kw.
4. (PillCam adj2 (colon* or "COLON 2" or COLON2)).ti,ab,kw.
5. ("second-generation colon capsule" or "second generation colon capsule" or "second-generation colonic capsule" or "second generation colonic capsule" or CCE-2 or CCE2).ti,ab,kw.
6. 1 or 2 or 3 or 4 or 5
7. exp intestine polyp/ or exp colorectal tumor/ or exp colorectal cancer/ or exp adenoma/
8. (polyp* or polypoid or polypoidal or adenoma* or neoplas* or carcinoma* or cancer* or tumor* or tumour* or lesion*).ti,ab,kw.
9. (colorectal adj2 (cancer* or carcinoma* or neoplas* or tumor* or tumour*)).ti,ab,kw.
10. (colon* adj2 (cancer* or carcinoma* or neoplas* or tumor* or tumour*)).ti,ab,kw.
11. (rectal adj2 (cancer* or carcinoma* or neoplas* or tumor* or tumour*)).ti,ab,kw.
12. (advanced adj2 (adenoma* or neoplas* or polyp*)).ti,ab,kw.
13. (serrated adj2 (lesion* or polyp* or adenoma*)).ti,ab,kw.
14. ((sessile adj1 serrated) adj2 (lesion* or polyp* or adenoma*)).ti,ab,kw.
15. 7 or 8 or 9 or 10 or 11 or 12 or 13 or 14
16. 6 and 15
17. limit 16 to yr="2004 - 2026"

S2.3 Ovid MEDLINE

1. (colon capsule endoscop* or colonic capsule endoscop* or colon capsule examination* or colonic capsule examination* or capsule colonoscop* or colon capsule* or colonic capsule* or colon video capsule* or colonic video capsule*).ti,ab,kw.
2. ((capsule endoscop* or video capsule*) adj3 (colon* or colorect*)).ti,ab,kw.
3. (capsule* adj3 (colonoscop* or colon* or colorect*)).ti,ab,kw.
4. (PillCam adj2 (colon* or "COLON 2" or COLON2)).ti,ab,kw.
5. ("second-generation colon capsule" or "second generation colon capsule" or "second-generation colonic capsule" or "second generation colonic capsule" or CCE-2 or CCE2).ti,ab,kw.
6. 1 or 2 or 3 or 4 or 5
7. exp Colonic Polyps/ or exp Adenoma/ or exp Colorectal Neoplasms/
8. (polyp* or polypoid or polypoidal or adenoma* or neoplas* or carcinoma* or cancer* or tumor* or tumour* or lesion*).ti,ab,kw.
9. (colorectal adj2 (cancer* or carcinoma* or neoplas* or tumor* or tumour*)).ti,ab,kw.
10. (colon* adj2 (cancer* or carcinoma* or neoplas* or tumor* or tumour*)).ti,ab,kw.
11. (rectal adj2 (cancer* or carcinoma* or neoplas* or tumor* or tumour*)).ti,ab,kw.
12. (advanced adj2 (adenoma* or neoplas* or polyp*)).ti,ab,kw.
13. (serrated adj2 (lesion* or polyp* or adenoma*)).ti,ab,kw.
14. ((sessile adj1 serrated) adj2 (lesion* or polyp* or adenoma*)).ti,ab,kw.
15. 7 or 8 or 9 or 10 or 11 or 12 or 13 or 14
16. 6 and 15
17. limit 16 to yr="2004 - 2026"

S2.4 Cochrane CENTRAL

#1 ("colon capsule endoscopy" OR "colonic capsule endoscopy" OR "colon capsule examination" OR "colonic capsule examination" OR "capsule colonoscopy" OR "colon capsule" OR "colonic capsule" OR "colon video capsule" OR "colonic video capsule"):ti,ab,kw
#2 ((capsule endoscop* OR video capsule*) NEAR/3 (colon* OR colorect*)):ti,ab,kw
#3 (capsule* NEAR/3 (colonoscop* OR colon* OR colorect*)):ti,ab,kw
#4 (PillCam NEAR/2 (colon* OR "COLON 2" OR COLON2)):ti,ab,kw
#5 ("second-generation colon capsule" OR "second generation colon capsule" OR "second-generation colonic capsule" OR "second generation colonic capsule" OR "CCE-2" OR CCE2):ti,ab,kw
#6 #1 OR #2 OR #3 OR #4 OR #5
#7 MeSH descriptor: [Colonic Polyps] explode all trees
#8 MeSH descriptor: [Adenoma] explode all trees
#9 MeSH descriptor: [Colorectal Neoplasms] explode all trees
#10 (polyp* OR polypoid OR polypoidal OR adenoma* OR neoplas* OR carcinoma* OR cancer* OR tumor* OR tumour* OR lesion*):ti,ab,kw
#11 (colorectal NEAR/2 (cancer* OR carcinoma* OR neoplas* OR tumor* OR tumour*)):ti,ab,kw
#12 (colon* NEAR/2 (cancer* OR carcinoma* OR neoplas* OR tumor* OR tumour*)):ti,ab,kw
#13 (rectal NEAR/2 (cancer* OR carcinoma* OR neoplas* OR tumor* OR tumour*)):ti,ab,kw

#14 (advanced NEAR/2 (adenoma* OR neoplas* OR polyp*)):ti,ab,kw
#15 (serrated NEAR/2 (lesion* OR polyp* OR adenoma*)):ti,ab,kw
#16 ((sessile NEAR/1 serrated) NEAR/2 (lesion* OR polyp* OR adenoma*)):ti,ab,kw
#17 #7 OR #8 OR #9 OR #10 OR #11 OR #12 OR #13 OR #14 OR #15 OR #16
#18 #6 AND #17
(Apply Cochrane Library filters: Trials/CENTRAL, publication year 2004–2026.)

S3. Eligibility criteria PICO

Table S1. Eligibility criteria (inclusion and exclusion).

Domain	Included	Excluded
Population	Adults undergoing CCE for any colorectal indication (screening, FIT+, surveillance, symptomatic, post-incomplete colonoscopy)	Populations selected on known polyps only, advanced CRC only, or small-bowel indications; paediatric
Intervention	Dedicated colon capsule endoscopy (CCE1 or CCE2 / PillCam COLON, OMOM CC100 and equivalent devices); panenteric capsule where colonic results were separately extractable	Gastric or small-bowel-only capsule; phantom/ex-vivo/animal; algorithm-only image datasets
Comparator	None required (single-arm proportion); CCE arm of comparative studies eligible	—
Outcome	Extractable per-patient any-polyp and/or size-threshold (≥6 mm or ≥10 mm) detection rate, with a numerator (patients with ≥1 colorectal polyp detected by CCE at the relevant threshold) and a denominator. The preferred denominator is all swallowed capsules; alternative analysed or complete-examination denominators must be identified.	Per-lesion / per-polyp / per-frame counts; sensitivity/specificity only; % without numerator; no polyp outcome
Study design	Prospective/retrospective cohort; CCE arm of RCT/comparative	Reviews, editorials, commentaries, HTAs, protocols, modelling/economic studies, surveys, case reports/series (n<10), and conference abstracts.
Timeframe/Language	2004–2026	—

S4. Risk of bias

Study	Q1	Q2	Q3	Q4	Q5	Q6	Q7	Q8	Q9	Overall
Schoofs 2006	?	?	–	+	+	+	+	+	+	MOD
Eliakim 2009	+	?	+	+	+	+	+	+	+	MOD
Sieg 2009	+	–	–	+	–	+	+	?	–	HIGH
Gay 2010	?	?	+	+	+	+	+	+	+	MOD
Pilz 2010	?	?	–	+	+	+	+	+	+	MOD
Sacher-Huvelin 2010	+	?	+	+	+	+	+	+	+	LOW
Herrerías-Gutiérrez 2011	?	+	+	+	–	+	?	+	–	HIGH
Spada 2011a	?	–	+	+	+	+	+	+	+	MOD
Spada 2011b	?	?	–	+	–	+	+	+	–	HIGH
Mussetto 2012	?	–	–	+	–	?	?	–	–	HIGH
Pioche 2012	?	?	+	+	–	+	+	+	–	HIGH
Holleran 2014	+	?	–	+	+	+	+	+	–	MOD
Spada 2014	?	+	+	+	–	+	+	+	–	MOD
Togashi 2015	–	?	–	+	+	+	?	+	+	HIGH
Brechmann 2016	?	?	–	+	–	+	+	+	–	HIGH
Morgan 2016	+	?	–	+	+	+	+	+	+	MOD
Romero-Vázquez 2016	?	?	+	+	+	+	?	+	+	MOD
Alvarez-Urturi 2017	+	?	–	+	–	+	+	+	–	MOD
Nogales 2017	?	+	+	+	+	+	?	+	+	MOD
Baltes 2018	+	+	–	+	+	+	+	+	+	LOW
Hussey 2018	?	?	–	+	+	+	?	?	+	HIGH
Parodi 2018	+	+	+	+	+	+	+	+	+	LOW
Voska 2019	+	?	+	+	+	+	+	+	+	LOW
Cash 2020	+	+	+	+	–	+	+	+	–	LOW
González-Suárez 2020	+	+	+	+	+	+	+	+	–	LOW
Nakaji 2020	–	+	+	+	–	+	?	+	–	HIGH
Pecere 2020	+	+	+	+	–	+	?	+	–	MOD
Benech 2021	?	+	+	+	–	+	+	+	–	MOD
Deding 2021 *	+	+	+	+	+	?	+	+	+	LOW
Hausmann 2021	?	–	+	+	+	+	+	+	+	MOD
Ismail 2021 *	+	–	–	+	–	+	+	+	–	HIGH
Semenov 2021	?	–	+	+	+	+	+	+	+	MOD
Takashima 2021	?	–	–	+	+	+	+	+	+	HIGH
Deding 2022 *	+	–	+	+	+	+	+	+	+	LOW
Semenov 2022	?	–	+	+	–	+	+	+	–	HIGH
MacLeod 2024a	?	?	+	+	+	+	?	+	+	MOD
Omori 2024	+	?	+	+	+	+	+	+	+	LOW
Benamouzig 2025	–	+	–	+	+	+	–	+	–	HIGH
MacLeod 2025	+	?	+	+	–	+	+	+	–	MOD
Semenov 2025	+	–	+	+	+	+	+	+	–	MOD
Turvill 2025	+	?	+	+	+	+	+	+	–	LOW
Agache 2026	?	+	+	+	+	+	+	+	–	MOD
Deding 2026 *	+	?	+	+	–	+	–	+	–	MOD
Marijnissen 2026	+	–	–	+	–	+	?	+	–	HIGH
Selnes 2026	+	–	+	+	–	+	+	+	–	MOD
Thorndal 2026	+	+	–	+	–	+	–	+	–	HIGH
Turvill 2026	?	–	+	+	+	+	+	–	–	HIGH
Xie 2026	+	?	+	+	+	+	+	+	+	LOW

Yes / Low · Unclear / Moderate · No / High · Q1 sampling frame · Q2 sampling method · Q3 sample size · Q4 subjects/setting · Q5 coverage · Q6 valid measure · Q7 reliable measure · Q8 statistics · Q9 response rate

Figure S1. Risk of bias — JBI critical-appraisal checklist for prevalence studies. Green = Yes / Low; amber = Unclear / Moderate; red = No / High. All 48 candidate reports were appraised independently by two reviewers before the final eligibility and overlap decision. Where the reviewers assigned the same overall rating that rating stood; the remainder were adjudicated item by item against the source reports (see S4.1). * marks the four reports excluded at that step (Deding et al. 2021, Deding et al. 2022, Deding et al. 2026, and Ismail et al. 2021), leaving the 44 retained reports/datasets shown in Table 1 (see S7). Studies are labelled by first author and publication year: Schoofs 2006 [8]; Eliakim 2009 [9]; Sieg 2009 [10]; Gay 2010 [11]; Pilz 2010 [12]; Sacher-Huvelin 2010 [13]; Herrerías-Gutiérrez 2011 [14]; Spada 2011a [15]; Spada 2011b [16]; Mussetto 2012 [17]; Pioche 2012 [18]; Holleran 2014 [19]; Spada 2014 [20]; Togashi 2015 [21]; Brechmann 2016 [22]; Morgan 2016 [23]; Romero-Vázquez 2016 [24]; Alvarez-Urturi 2017 [25]; Nogales 2017 [26]; Baltes 2018 [27]; Hussey 2018 [28]; Parodi 2018 [29]; Voska 2019 [30]; Cash 2020 [31]; González-

Suárez 2020 [32]; Nakaji 2020 [33]; Pecere 2020 [34]; Benech 2021 [35]; Hausmann 2021 [36]; Semenov 2021 [37]; Takashima 2021 [38]; Semenov 2022 [39]; MacLeod 2024a [40]; Omori 2024 [41]; Benamouzig 2025 [42]; MacLeod 2025 [43]; Semenov 2025 [44]; Turvill 2025 [45]; Agache 2026 [46]; Marijnissen 2026 [47]; Selnes 2026 [48]; Thorndal 2026 [49]; Turvill 2026 [50]; Xie 2026 [51]; Deding 2021 [72]; Deding 2022 [73]; Deding 2026 [74]; Ismail 2021 [75].

S4.1 Reconciliation of the two risk-of-bias appraisals

Disagreements between the two independent reviewers were resolved by reassessing individual JBI items against prespecified criteria and the source publication. Sample-size adequacy (Q3) was based on the precision of the study-specific detection estimate, with a 95% CI half-width greater than 10 percentage points considered inadequate. For coverage and response rate (Q5 and Q9), assessment was based on the analytical denominator. Studies that excluded incomplete examinations were considered at greater risk of bias, whereas those retaining incomplete examinations in the denominator were not penalised twice for the same limitation. All other items were assessed directly from the reported methods. Final overall ratings were author-defined from the reconciled item-level assessments; the JBI checklist itself does not define low/moderate/high overall categories, which by convention JBI leaves to reviewers. The item-level reconciliation criteria used in this review are stated above. Overall categories were derived from the reconciled items: an item scored no or unclear was counted as not met, and overall risk was low with 0–1, moderate with 2–3, and high with 4 or more unmet items. This mapping reproduced 18 of the 22 overall ratings on which both reviewers had independently agreed; agreed ratings stood (22 reports), and the thresholds were applied to the reconciled items where reviewers disagreed (21 reports; one further query on publication type was resolved by consensus). Applied to every report, the thresholds reclassified five of 44 reports by one category (Eliakim 2009 moderate to low; Turvill 2025 and Cash 2020 low to moderate; Takashima 2021 high to moderate; Alvarez-Urturi 2017 moderate to high), and excluding high-risk studies then gave a PDR of 58.0% (k=21; Table S5).

S5. Meta-analysis results

The primary analysis included 31 independent studies and used a random-intercept binomial-normal generalised linear mixed model with a logit link, fitted using metafor::rma.glmm. Robustness of the pooled estimate was assessed using a Freeman–Tukey transformed random-effects model with REML estimation, leave-one-out analyses, exclusion of reconstructed counts, and exclusion of high-risk-of-bias studies. A broader sensitivity analysis included 37 datasets, incorporating six additional non-primary datasets. Because some of these datasets were not statistically independent, this analysis was used only to assess robustness and was not treated as an independent-study pool (S6.1). Formal funnel-plot asymmetry testing was not used because substantial clinical heterogeneity and the conditions required for meaningful asymmetry testing were not met; reporting bias was assessed qualitatively. As post hoc descriptive checks, smaller (<100 participants) and larger studies were compared and logit PDR was regressed on $1/\sqrt{n}$; t-based GLMM confidence intervals without a Knapp–Hartung standard-error adjustment gave 53.2% (95% CI 45.9–60.4); Hartung–Knapp intervals for the Freeman–Tukey/REML model gave 52.8% (45.8–59.8).

Table S2. Pooled per-patient detection rates (any-polyp, ≥6 mm, ≥10 mm), with heterogeneity.

Outcome	k	GLMM PDR	95% CI	95% PI	Crude	FTT/REML
Any-polyp (primary pool: de-duplicated + uniform definition + n≥10)	31	53.2%	46.1–60.1	19–85	51.7%	52.8%
≥6 mm	23	32.7%	25.4–41.0	8–74	35.5%	33.3%
≥10 mm	16	17.7%	12.7–24.1	4–51	20.3%	18.4%

S5.1 Exploratory analysis by referral pathway (primary pool; omnibus meta-regression p<0.001)
Table S3. Any-polyp detection rate by exploratory referral pathway (primary pool; registered ≥80% single-indication rule).

**Referral pathway**	k	GLMM PDR	95% CI; 95% PI; I^2	Crude
**FIT-positive screening**	4	81.2%	72.2–87.8; 47–96%; 79%	79.7%
**Screening without preceding positive FIT**	3	39.9%	31.5–49.0; 15–72%; 76%	42.1%
**Symptomatic**	2	53.0%	39.1–66.4; —; 99%	46.6%
**Post-incomplete colonoscopy**	4	49.4%	35.2–63.6; 15–84%; 66%	46.0%
**Post-polypectomy surveillance**	1	65.9% (single study; not pooled)	—	65.9%
**Mixed / unclassifiable**	14	49.7%	40.7–58.8; 18–81%; 89%	47.3%
**Colonoscopy declined**	1	40.0% (single study; not pooled)	—	40.0%
**Other: anticipated difficult colonoscopy**	1	45.7% (single study; not pooled)	—	45.7%
**Other: post-diverticulitis follow-up**	1	32.8% (single study; not pooled)	—	32.8%

Sensitivity analysis. Under the source-level classification used before the registered rule was applied (MacLeod 2025 and Romero-Vázquez 2016 as mixed, Nakaji 2020 and Thorndal 2026 as mixed/other, Mussetto 2012 as screening), PDR was 81.2% in FIT-positive screening (k=4), 40.8% (29.5–53.2) in screening without FIT (k=3), and 48.7% (41.2–56.2) in mixed/other cohorts (k=18); pathway accounted for 51% of between-study variance, and the pathway-adjusted CCE2 OR was 1.61 (0.97–2.69). Prediction intervals were not reported for the two-study stratum. Threshold sensitivity (post hoc): with a 70% threshold, Brechmann 2016 (76% symptoms or anaemia) and Xie 2026 (70% symptoms) became symptomatic, giving 47.8% (38.1–57.8; k=4) in symptomatic and 51.2% (k=12) in mixed cohorts; with 75%, only Brechmann 2016 moved (symptomatic 49.9%, k=3); with 90%, Romero-Vázquez 2016 (81% screening) became mixed, giving 40.6% (27.9–54.7; k=2) in screening without FIT. FIT-positive screening (81.2%) and post-incomplete

colonoscopy (49.4%) were unchanged at every threshold because all those cohorts had a single indication; between 70% and 90%, at most 2 of 31 cohorts changed stratum.

S5.2 Meta-regression (logit GLMM, on the primary pool)

Table S4. Meta-regression of candidate moderators (univariable unless stated).

Moderator	Estimate (95% CI)	p	Interpretation
**Referral pathway (omnibus)**	—	<0.001	Exploratory; registered ≥80% rule; pseudo-R^2 55%; residual I^2 92%
**Capsule generation (CCE2 vs CCE1), unadjusted**	OR 2.17 (1.19–3.95)	0.012	Exploratory; confounded by pathway/era
**Capsule generation, adjusted for referral pathway**	OR 1.67 (0.95–2.92)	0.073	Attenuated; no independent effect established
**Publication year**	—	0.25	No clear association
**Denominator convention**	—	0.61	No clear association
**Capsule generation, adjusted for publication year (post hoc)**	OR 2.16 (0.95–4.92)	0.067	Point estimate unchanged; imprecise; collinear with era
**Capsule generation within the mixed stratum (post hoc; 7 CCE1, 6 CCE2)**	OR 1.62 (0.77–3.38)	0.20	No clear association
**Adequate bowel preparation, per 10 percentage points (k=26)**	OR 1.08 (0.89–1.31)	0.46	No clear association; definitions varied
**Complete examinations, per 10 percentage points (k=31)**	OR 0.96 (0.74–1.24)	0.75	No clear association; definitions varied
**Region: Asia/Middle East vs Europe (k=5 vs 26)**	OR 1.24 (0.57–2.68)	0.59	No clear association
**Mean or median age, per 10 years (k=30)**	OR 1.87 (1.15–3.04)	0.012	Attenuated after adjustment for pathway: OR 1.48 (0.98–2.23), p=0.059
**Men, per 10 percentage points (k=29)**	OR 1.10 (0.89–1.37)	0.37	No clear association

Figure S2. Forest plot of the pooled per-patient CCE detection rate for polyps ≥10 mm. Studies are labelled by first author and publication year: Eliakim 2009 [9]; Spada 2011a [15]; Mussetto 2012 [17]; Spada 2014 [20]; Togashi 2015 [21]; Brechmann 2016 [22]; Morgan 2016 [23]; Parodi 2018 [29]; Voska 2019 [30]; Cash 2020 [31]; MacLeod 2024a [40]; MacLeod 2025 [43]; Turvill 2025 [45]; Marijnissen 2026 [47]; Selnes 2026 [48]; Turvill 2026 [50].

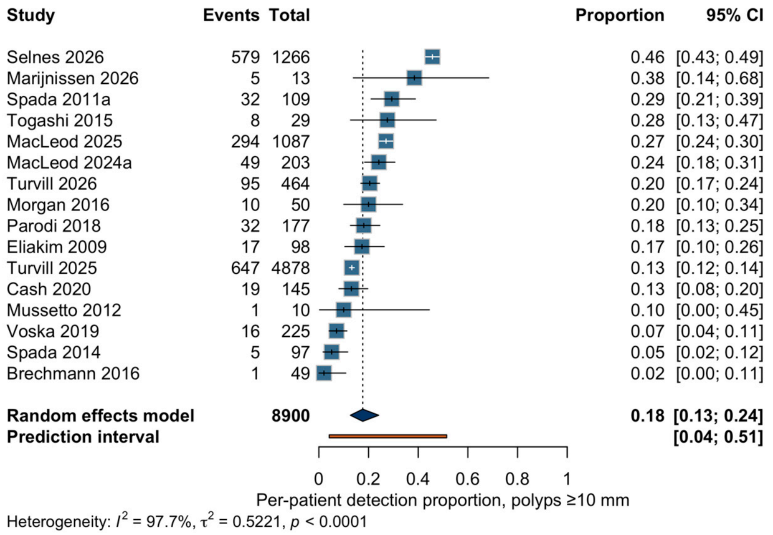

S6. Sensitivity analyses (any-polyp)

Table S5. Sensitivity analyses (any-polyp).

Analysis	k	PDR	Note
Primary (de-dup + uniform def. + n≥10, GLMM)	31	53.2%	Primary outcome
Freeman–Tukey / REML	31	52.8%	Similar pooled estimate
Leave-one-out (range)	30 per iteration	51.4–54.3%	no single study changed conclusion
Expanded 37-dataset sensitivity analysis	37	51.7%	All 37 datasets in Table 1 with an extractable any-polyp numerator, including the six non-primary datasets; not an independent-study pool
Exclude reconstructed (TP+FP) studies	26	51.3%	Five reconstructed-count studies are present in the 31-study primary pool; two further reconstructed-count reports lie outside that pool
Exclude high-risk-of-bias studies	21	55.8%	RoB does not drive the result
Exclude high-risk-of-bias studies, score thresholds applied to every study (post hoc)	21	58.0%	Thresholds reclassified 5 of 44 reports by one category (S4.1); with high risk defined as ≥3, ≥5 or ≥6 unmet items, PDR was 51.2% (k=12), 54.8% (k=26) and 53.5% (k=30)
All-swallowed or analysable-video denominator only (post hoc)	23	54.3%	95% CI 48.0–60.5

Complete or adequate-examination denominator only (post hoc)	7	49.9%	95% CI 29.2–70.7; imprecise; cannot exclude a meaningful difference
Studies with <100 participants (post hoc, descriptive)	15	51.1%	Logit PDR on 1/√n: p=0.89; low power under this heterogeneity
Studies with ≥100 participants (post hoc, descriptive)	16	54.8%	—

S6.1 Derivation of the primary any-polyp pool
Table 1 included 37 datasets with an extractable per-patient any-polyp numerator, comprising 12,013 participants, all of which were represented in the analytic dataset. Romero-Vázquez et al. 2016 (60/161) and Hausmann et al. 2021 (52/153) were initially excluded, but both numerators were subsequently verified manually against the source publications, where they were directly reported using a compatible any-polyp definition, and were therefore reinstated.

Six datasets were excluded from the primary analysis because of definitional non-equivalence, limited provenance, or cohort overlap. Agache et al. combined colonic and small-bowel lesions; Benamouzig et al. used a panenteric capsule and reported only diminutive or small polyps in a suspected irritable-bowel-syndrome population; Semenov et al. 2025 drew on the same institutional database as Semenov et al. 2021 over overlapping periods; González-Suárez et al. provided a neoplasia-only estimate reconstructed from diagnostic-accuracy measures rather than a directly reported any-polyp count; Semenov et al. 2022 overlapped with the same-centre Semenov et al. 2021 cohort, which was retained as the representative dataset; and Pioche et al. reported a composite significant-lesion outcome, defined as a polyp larger than 5 mm or at least three polyps, which was not definitionally comparable with the primary outcome. For Pioche et al., the 26/100 estimate shown in Table 1 was reconstructed during source verification by subtracting non-polyp lesions from the reported diagnostic yield of 36 among 100 analysable examinations and was therefore considered provenance-limited.

The primary analysis consequently included 31 independent studies comprising 11,310 participants. All six non-primary datasets were retained in an expanded 37-dataset sensitivity analysis, which produced a pooled estimate of 51.7%, similar to the primary estimate of 53.2%. Accordingly, the expanded 37-dataset analysis represents all datasets with an extractable per-patient any-polyp numerator, but should not be interpreted as a pool of 37 independent studies. The disposition of all 44 retained studies is given in Table S10.

S7. Overlapping-cohort handling (by recruitment period)
Recruitment periods, study centres, eligibility criteria, and trial identifiers were compared for all potentially overlapping study clusters. CareForColon2015, Denmark. Selnes et al. 2026, Deding et al. 2026, Deding et al. 2022, and Deding et al. 2021 arose from the same FIT-positive trial and covered overlapping recruitment between August 2020 and December 2022. Selnes et al. 2026,

the verified full-cohort report, was retained, and the three Deding publications were excluded to prevent double-counting.

ScotCap, Scotland. MacLeod et al. 2024a reported the NHS Highland evaluation conducted from June 2019 to May 2020, whereas MacLeod et al. 2025 reported the national ScotCap registry from August 2020 to August 2021. These were consecutive, non-overlapping cohorts and were therefore retained as separate studies. MacLeod et al. 2025 contributed to all relevant outcomes. MacLeod et al. 2024a did not report a verifiable per-patient any-polyp estimate and contributed only to the size-stratified analyses, for which source-verified counts were available for polyps measuring at least 6 mm and at least 10 mm.

Semenov, Tallaght–Trinity, Dublin. Semenov et al. 2021, 2022, and 2025 drew from the same institutional database over overlapping periods. Only Semenov et al. 2021 was retained in the primary any-polyp analysis because it provided the verified outcome definition and was the only report contributing source-verified size-stratified data. The later reports were retained only in the expanded sensitivity analysis. Their inclusion had negligible effect on the pooled estimate. Ismail et al. 2021, from the same centre, reported 66 paired capsule and colonoscopy examinations drawn from a recruitment window overlapping that of Semenov et al. 2021 (186 examinations), which was retained as the representative cohort. Ismail et al. 2021 was in any case not eligible for either analysis: its only size-threshold outcome used a composite definition (more than three lesions or a polyp larger than 6 mm; 8/66) that cannot be reduced to a per-patient ≥6 mm count, and its any-polyp figure (23/66) was derived from a reported percentage rather than stated directly. It therefore appears in Figure S1, which shows all 48 appraised candidate reports, but not in Table 1.

S8. GRADE certainty of evidence

Certainty was rated using a GRADE-informed framework adapted from GRADE Working Group guidance for prognostic event rates [76], applied to the pooled proportions as registered. Each body of evidence started at high certainty and was rated down for risk of bias (substantial weight from high-risk studies), inconsistency (mainly the width of the 95% prediction interval, with I^2 as supporting information), indirectness (mixed pathways and outcome-definition variants), imprecision (confidence-interval width and numbers of studies and events), and publication bias. Publication bias was suspected for every outcome but not rated down, because the large registries and service evaluations included are likely to be published regardless of their results. Even so, missing studies cannot be ruled out, and the small-study checks were inconclusive (Tables S5 and S6).

Table S6. GRADE: certainty of evidence.

Outcome	RoB	Inconsistency	Indirectness	Imprecision	Pub. bias	Certainty
Any-polyp (primary)	serious (−1)	serious (−1; I^2=96%, PI 19–85, partly associated with pathway, pseudo-R^2 55%)	not serious	not serious (k=31, N=11,310, tight CI)	suspected; not rated down (large registries and service evaluations likely to be published; small-study comparison inconclusive)	Low
≥6 mm	serious (−1)	very serious (−2; PI 8–74, I^2=98%)	serious (−1; "significant polyp" variants)	not serious	suspected; not rated down (large cohorts likely to be published; small-study comparison inconclusive)	Very low
≥10 mm	serious (−1)	very serious (−2; PI 4–51)	serious (−1)	serious (−1; sparse, low events)	suspected; not rated down (large cohorts likely to be published; small-study comparison inconclusive)	Very low
By-referral-pathway strata	serious	serious	serious	very serious (k=1–14)	suspected; not rated down (as for any-polyp)	Very low (exploratory)

S9. Capsule generation, review timeline, protocol deviations, analytic pools, and study-level characteristics

Table S7. Capsule generation by referral pathway (primary pool, registered classification), with median publication year.

Referral pathway	CCE1	CCE2	OMOM CC100	Unclear	Total
**FIT-positive screening**	0	4	0	0	4
**Screening without preceding positive FIT**	1	1	0	1	3
**Symptomatic**	0	2	0	0	2
**Post-incomplete colonoscopy**	0	3	0	1	4
**Post-polypectomy surveillance**	0	1	0	0	1
**Colonoscopy declined**	1	0	0	0	1
**Other (two single studies)**	0	1	0	1	2
**Mixed / unclassifiable**	7	6	1	0	14
**Total**	9	18	1	3	31
**Median publication year**	2010	2021	2026	2018	—

CCE2 versus CCE1 (27 studies): unadjusted OR 2.17 (1.19–3.95); pathway-adjusted 1.67 (0.95–2.92); year-adjusted 2.16 (0.95–4.92); mixed stratum only 1.62 (0.77–3.38). Generation, pathway, and era are collinear.

Table S8. Timeline of protocol registration, searching, and analysis.

Date	Event
**26 July 2026**	Full protocol finalised
**26–28 July 2026**	Preliminary database searches (PubMed 26 July; Ovid MEDLINE and Embase 28 July)
**July–August 2026**	Title/abstract screening, full-text assessment, and data extraction
**29 July 2026**	Protocol (version 3.0) submitted to PROSPERO; definitive full search of all four databases run
**19 August 2026**	PROSPERO registration approved (CRD420261464937)

Table S9. Deviations from the registered protocol (PROSPERO CRD420261464937).

Registered plan	What was done	Reason and impact
**Freeman–Tukey transformation with DerSimonian–Laird as primary model; GLMM as sensitivity analysis**	Binomial-normal GLMM as primary model; Freeman–Tukey/REML as sensitivity analysis	Study sizes ranged from 10 to 4,878, and double-arcsine back-transformation is unstable when sizes vary widely. Estimates agreed (53.2% vs 52.8%).
**Hartung–Knapp–Sidik–Jonkman confidence intervals**	Wald-type GLMM intervals reported; unadjusted t-based GLMM intervals and Hartung–Knapp Freeman–Tukey/REML intervals also computed	Negligible impact: 45.9–60.4% vs 46.1–60.1% (S5).

**Contour-enhanced funnel plot and Egger test when ≥10 studies**	Not performed as registered; a descriptive regression of logit PDR on 1/√n and a comparison by study size were performed as descriptive checks	Extreme heterogeneity and a single-proportion outcome make asymmetry tests unreliable. Both checks were inconclusive (p=0.89; Table S5) and are not evidence against small-study effects.
**ROB-ME assessment and registry scan for missing evidence**	Not performed	Reporting bias was assessed qualitatively and considered within the GRADE publication-bias domain (S8).
**Overall estimate presented as secondary to indication-stratified estimates**	Overall estimate reported as the headline; pathway estimates reported as exploratory	Fourteen of 31 cohorts could not be assigned to a single pathway, and several strata contained one to four studies.
**Indication strata including iron-deficiency anaemia, colonoscopy contraindicated or declined, and other**	Registered ≥80% rule applied; no cohort met the rule for iron-deficiency anaemia; one cohort classed as colonoscopy declined; two unrelated single cohorts classed as other and reported individually	Pooling unrelated single cohorts would not be interpretable.
**≥80% single-indication rule for assigning cohorts to strata**	Applied; an earlier source-level classification was replaced	Five cohorts reassigned (Section 3.6); the source-level classification is reported as a sensitivity analysis with a similar pattern (Table S3).
**Subgroups by bowel-preparation adequacy and completion-rate category**	Analysed as continuous study-level moderators (per 10 percentage points)	Avoids arbitrary category cut-offs; no association found (Table S4).
**Moderators: colonic transit time, reading time, reader experience or case volume, FIT threshold**	Not analysed	Reported inconsistently or by too few studies (FIT threshold available for 3 of 4 FIT-positive cohorts; reader experience quantified in 5 of 31 studies).
**Additional outcome: advanced-neoplasia detection**	Not synthesised	Advanced neoplasia requires histological verification, available only in paired-colonoscopy subsets with heterogeneous definitions, and falls outside a visual-yield synthesis.
**Additional outcome: per-patient sensitivity and specificity from paired colonoscopy**	Not synthesised	Outside the yield estimand and already synthesised in recent reviews, including references 67 and 68 of the main text.
**Influence diagnostics including Baujat plots**	Leave-one-out analyses only	Leave-one-out estimates are reported for every pooled outcome.
**Eligible devices: PillCam COLON 1 and 2 and equivalent colon capsules**	One panenteric capsule study included where colonic results were separately extractable	The one such study (Benamouzig 2025) was excluded from the primary pool and contributes only to the expanded 37-dataset sensitivity analysis.
**Primary denominator of all ingested (swallowed) capsules; diagnostically adequate examinations as the sensitivity denominator**	Seven studies reporting only complete or adequate-examination denominators were included in the primary pool	Restricting the pool to all-swallowed or analysable-video denominators (k=23) gave 54.3% (95% CI 48.0–60.5), compared with 53.2% (Table S5).
**Full-text retrieval restricted to open-access sources**	All full texts were obtained through institutional library access	Removes the anticipated open-access selection bias; the open-access restriction was not applied in GRADE or the reporting-bias assessment.
**Summary-of-findings rating for each indication subgroup**	A single certainty rating covering all pathway strata	The strata contain one to 14 studies with shared limitations and would each be rated very low (Table S6).

Table S10. Derivation of the analytic pools from the 44 retained studies.

Step	Studies	Participants	Studies and reason
**Retained after full-text screening and overlap resolution**	44	12,754	All studies in Table 1 of the main text
**Size-threshold outcomes only (no any-polyp**	7	741	Spada 2011b (40), Spada 2014 (97), Togashi 2015 (29),

**numerator)**			Morgan 2016 (50), Parodi 2018 (177), Cash 2020 (145), MacLeod 2024a (203)
**Extractable any-polyp numerator (expanded sensitivity pool)**	37	12,013	—
**Excluded from primary pool: definition**	1	119	Agache 2026: colonic and small-bowel lesions combined
**Excluded from primary pool: definition**	1	29	Benamouzig 2025: panenteric capsule; only diminutive or small polyps reported
**Excluded from primary pool: overlap**	1	126	Semenov 2025: same institutional database as Semenov 2021, over overlapping periods
**Excluded from primary pool: definition and reconstructed numerator**	1	145	González-Suárez 2020: neoplasia-only count reconstructed from diagnostic-accuracy measures
**Excluded from primary pool: overlap**	1	184	Semenov 2022: overlapping cohort with Semenov 2021, which was retained
**Excluded from primary pool: definition and provenance**	1	100	Pioche 2012: composite significant-lesion outcome (polyp >5 mm or ≥3 polyps); numerator derived from the reported diagnostic yield (provenance-limited)
**Primary any-polyp pool**	31	11,310	Independent studies with a uniform any-polyp definition and n≥10

Table S11. FIT-positive cohorts: FIT threshold, programme, and route to CCE.

Study	FIT threshold	Assay	Programme	Ages	Route to CCE	PDR denominator
**Holleran 2014**	>100 ng Hb/mL in either of two samples (equivalent to 20 μg Hb/g)	OC-Sensor	Population-based biennial pilot, Dublin, Ireland (round 2)	50–75	Random subset of FIT-positive participants willing to undergo CCE and colonoscopy	All 62 who swallowed the capsule
**Pecere 2020**	Not reported	Not reported	Organised regional programmes, Italy (3 centres) and Tenerife, Spain	50–69	Consecutive FIT-positive screenees referred for first colonoscopy	Complete CCE and colonoscopy (178 of 222 enrolled)
**Marijnissen 2026**	47 μg Hb/g	Not reported	Dutch national screening programme	55–75	Unable or unwilling to have colonoscopy, or after incomplete colonoscopy; 13 of 169 such FIT-positive participants underwent CCE	All 13

**Selnes 2026**	>20 µg Hb/g	Not reported	Danish national programme (CareForColon2015 trial)	50–74	Chose CCE in the trial intervention arm	Complete CCE with full covariates (1,266 of 2,031)
**González-Suárez 2020 (not in primary pool)**	≥20 µg Hb/g	Not reported	Organised programme, Barcelona, Spain	50–69	Randomised to CCE or CT colonography	Intention-to-screen CCE group (145)

Values were extracted from the source reports with verbatim quotations (Table S13).

Table S12. Study-level indication mix, denominator, bowel preparation, completion, reader qualification, age, and sex (primary pool).

Study	Pathway (indication mix)	Denominator	Cleansing scale	Adequate preparation	Completion	Reader qualification	Age (years)	Men
**Holleran 2014**	FIT-positive screening (100%)	All swallowed or analysable video	unvalidated 4-grade form	91.9%	74.2%	Consultant gastroenterologist trained in CCE	62.5	55%
**Marijnissen 2026**	FIT-positive screening (100%; unable or unwilling to have colonoscopy, or incomplete colonoscopy)	All swallowed or analysable video	4-grade unnamed	38.5%	69.2%	Two researchers qualified through CCE e-learning	67 (median)	92.3%
**Pecere 2020**	FIT-positive screening (100%)	Complete or adequate	dichotomous unnamed	88.2%	87.7%	Two endoscopists per centre with documented capsule experience	61	56.3%
**Selnes 2026**	FIT-positive screening (100%)	Complete or adequate	Leighton-Rex	NR	69.6%	Trained CCE readers	NR (bands only)	54.3%
**Alvarez-Urturi 2017**	Screening, familial risk (100%)	Complete or adequate	4-grade unnamed	60.7%	86.3%	Two physicians with CCE expertise	48.6	45.1%
**Romero-Vázquez 2016**	Screening (81%; diarrhoea 10%, IDA 6%, incomplete colonoscopy 3%)	Other	4-grade unnamed	78.3%	86.1%	NR	56.4	50.3%
**Voska 2019**	Screening, average risk (100%)	All swallowed or analysable video	4-grade A-D	89.8%	89.3%	Small-bowel capsule experience and CCE reading training	59	53%
**MacLeod 2025**	Symptomatic (96%; surveillance 4%)	All swallowed or analysable video	Leighton-Rex	59.2%	57%	Two consultant gastroenterologists trained in CCE	60 (median)	41.7%
**Turvill 2025**	Symptomatic (100%; FIT ≤100 µg Hb/g)	All swallowed or analysable video	CC-CLEAR	74%	74%	Certified online CCE training course	60	45%
**Baltes 2018**	Post-incomplete colonoscopy (100%)	All swallowed or analysable video	4-grade unnamed	66.7%	64.9%	>25 CCE, >100 small-bowel capsule, >1,000 colonoscopies	66	40.5%
**Hussey 2018**	Post-incomplete colonoscopy (100%)	All swallowed or analysable video	Leighton-Rex form	NR	76%	Physician experienced in CCE	57	36%
**Nogales 2017**	Post-incomplete colonoscopy (100%)	All swallowed or analysable video	Leighton-Rex	74%	71.9%	Local readers at each centre; experience NR	58.1	32.3%
**Takashima 2021**	Post-incomplete colonoscopy (100%)	All swallowed or analysable	Aronchick	70.6%	88.2%	NR	59.5	17.6%

		video						
**Turvill 2026**	Post-polypectomy surveillance (100%)	All swallowed or analysable video	CC-CLEAR	73%	78.4%	Certified online CCE training course	62	59%
**Mussetto 2012**	Colonoscopy declined (all FOBT-positive and declined colonoscopy)	All swallowed or analysable video	unnamed	80%	60%	NR	57	30%
**Nakaji 2020**	Other (anticipated difficult colonoscopy 91%, failed colonoscopy 9%)	Complete or adequate	NR	NR	85%	Certified capsule technician and supervising physician	62	52%
**Thorndal 2026**	Other (post-diverticulitis follow-up, 100%)	All swallowed or analysable video	Leighton-Rex	88.5%	83.6%	Single expert with >4,000 CCE readings	60	36.1%
**Benech 2021**	Mixed (contraindication to colonoscopy 45%, incomplete colonoscopy 32%, refusal 21%)	All swallowed or analysable video	Leighton-Rex	69.2%	64.2%	>300 capsule readings and two-day CCE training	70 (median)	NR
**Brechmann 2016**	Mixed (symptoms or anaemia 76%, screening 18%, other 6%)	Complete or adequate	4-grade (Van Gossum)	38%	60%	Reviewing physicians; experience NR	59.5	48%
**Eliakim 2009**	Mixed (personal/family history 34%, screening 32%, bleeding or FOBT-positive 21%, symptoms 17%, IDA 3%)	All swallowed or analysable video	4-grade unnamed	78%	81%	Five blinded investigators with small-bowel capsule experience	49.8	66%
**Gay 2010**	Mixed (personal/family history 45%, symptoms 34%, screening 13%, other 8%)	All swallowed or analysable video	4-grade unnamed	81.7%	90.5%	Single investigator; experience NR	55	52.3%
**Hausmann 2021**	Mixed (symptoms 47%, screening 36%, IBD 7%, other 9%)	All swallowed or analysable video	Leighton 4-grade	80.1%	68.9%	Highly experienced investigators	57 (median)	51.6%
**Herrerías-Gutiérrez 2011**	Mixed (screening 61%, post-polypectomy 17%, symptoms or anaemia 17%, incomplete colonoscopy 5%)	Complete or adequate	4-grade unnamed	65.7%	93.1%	One physician; experience NR	52.2	53.5%
**Omori 2024**	Mixed (FIT-positive 39%, upper-GI screening 25%, polyp follow-up 22%, other 14%)	All swallowed or analysable video	Leighton-Rex	91%	88.8%	Trained colonoscopists with >20 CCE interpretations	66 (median)	73%
**Pilz 2010**	Mixed (screening 41%, symptoms 46%, family history or surveillance 13%)	All swallowed or analysable video	3-point departmental	NR	64.3%	Two departmental gastroenterologists	60	60.7%
**Sacher-Huvelin 2010**	Mixed (increased risk by	All swallowed or analysable	4-grade unnamed	52%	91.3%	One endoscopist per centre after one-day CCE	60	NR

	personal/family history 69%, average-risk screening 30%)	video				training		
**Schoofs 2006**	Mixed (screening 41%, symptoms 59%)	All swallowed or analysable video	4-grade (MST)	90%	83.3%	One physician; no prior CCE reading experience	56	36.6%
**Semenov 2021**	Mixed (polyp surveillance 52%, symptoms 23%, incomplete colonoscopy 15%, anaemia 10%)	All swallowed or analysable video	validated 4-grade (overall impression)	91.4%	77.4%	Trained CCE readers	60	44%
**Sieg 2009**	Mixed (screening 50%, FOBT-positive 45%, familial risk 5%)	Complete or adequate	4-grade unnamed	NR	94.7%	Two blinded investigators; qualification NR	56	83.3%
**Spada 2011a**	Mixed (personal history 44%, change in bowel habit 23%, screening 21%, bleeding 20%; more than one allowed)	All swallowed or analysable video	4-grade unnamed	81%	88%	Investigators with small-bowel capsule experience, trained in CCE	60	61.5%
**Xie 2026**	Mixed (symptoms 70%, screening 26%, polyp history 4%)	All swallowed or analysable video	Leighton binary	96.3%	91.2%	>10 years' experience; >200 capsule readings per year	45.2	54.4%

Pathway assigned with the registered ≥80% single-indication rule. Adequate preparation and completion are given as defined by each study; NR, not reported. Age is the mean, or the median where only that was reported. Verbatim source quotations for every value are in Table S13.
